# Automated optogenetic feedback control for precise and robust regulation of gene expression and cell growth

**DOI:** 10.1038/ncomms12546

**Published:** 2016-08-26

**Authors:** Andreas Milias-Argeitis, Marc Rullan, Stephanie K. Aoki, Peter Buchmann, Mustafa Khammash

**Affiliations:** 1 Department of Biosystems Science and Engineering, ETH Zurich, Basel 4058, Switzerland

## Abstract

Dynamic control of gene expression can have far-reaching implications for biotechnological applications and biological discovery. Thanks to the advantages of light, optogenetics has emerged as an ideal technology for this task. Current state-of-the-art methods for optical expression control fail to combine precision with repeatability and cannot withstand changing operating culture conditions. Here, we present a novel fully automatic experimental platform for the robust and precise long-term optogenetic regulation of protein production in liquid *Escherichia coli* cultures. Using a computer-controlled light-responsive two-component system, we accurately track prescribed dynamic green fluorescent protein expression profiles through the application of feedback control, and show that the system adapts to global perturbations such as nutrient and temperature changes. We demonstrate the efficacy and potential utility of our approach by placing a key metabolic enzyme under optogenetic control, thus enabling dynamic regulation of the culture growth rate with potential applications in bacterial physiology studies and biotechnology.

## Abstract (web summary)

Optogenetics has emerged as a promising means to achieve gene expression control in bioprocess engineering, but current systems cannot respond to fluctuations in growth conditions. Here the authors overcome this limitation and develop an automated optogenetic feedback control system for precise and robust control of protein production in *E. coli*.

## Graphical abstract

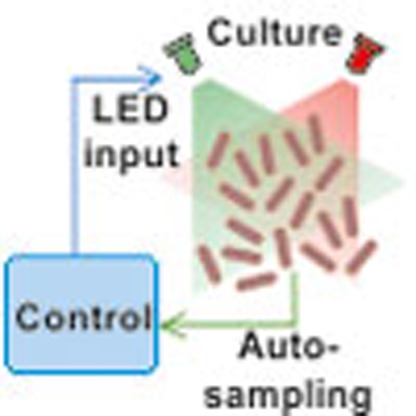

Optogenetic manipulation of biological systems holds the promise to revolutionize many areas of biology and biotechnology^1^. Using light-sensitive proteins and domains, light enables the rapid, targeted, low-cost and precise spatiotemporal modulation of protein function with low to no toxicity, while avoiding the pleiotropic effects of small-molecule inducers. These features have led to an explosive increase in the number of optogenetic applications in recent years^2^,^3^,^4^,^5^,^6^,^7^,^8^.

A key emerging optogenetic application is the control of gene expression. Such control may be achieved in an open-loop manner by administering light-induced perturbations to a given system with the goal of achieving a prescribed expression profile. State-of-the-art work in this area^9^ involved the use of a finely tuned mathematical model obtained through a long characterization process and recalibrated daily. While such open-loop operation is effective for parts characterization, as nicely demonstrated in Olson *et al*.^9^, the general applicability of this approach in biotechnology is ultimately limited by the fact that the model is only accurate for one specific culture condition, and any alterations or slight disturbances to cultures during the course of an experiment would result in inaccurate tracking. Precision, robustness and repeatability are thus restricted by day-to-day variability in cellular behaviour, changes in the cellular environment, and the typically limited quantitative understanding of the open-loop controlled system. Overcoming these challenges promises to unlock the huge potential of optogenetics for biotechnology applications. An effective and feasible technology for achieving this is *in silico* automatic feedback control^10^. This involves measuring the system output in real-time, comparing it against a desired tracking objective, and feeding the difference to a dynamic control system, which in turn uses it to compute the necessary adjustments of the system input. Automatic feedback control of cell populations has been implemented^11^,^12^,^13^ with promising results using microfluidics. However, while microfluidic approaches are well-suited for high-throughput analysis of single-cell behaviour as well as biomedical diagnostics, promising biotechnological applications of optogenetics, such as control of metabolic activity in microbial production strains^14^,^15^, require the use of large-volume liquid cell cultures.

In previous work^16^, we introduced feedback control for a red/far-red light system in yeast liquid cultures. While the implemented control scheme served as a proof-of-concept study of the applicability of feedback, tracking accuracy was rather modest and the controller was incapable of robust, precise regulation in the face of external perturbations and day-to-day variability. The feedback control also relied crucially on the experimenter manually acquiring samples and applying the necessary control inputs to the system—a tedious, repetitive and error-prone task that also limited the maximum measurement and input application frequency.

Here, we significantly improve on these results by presenting a completely automatic system capable of long-term optical feedback control of gene expression in continuous liquid cultures. We use a light-switchable cyanobacterial two-component system in *Escherichia coli*^9^,^17^ (Fig. 1a), consisting of the sensor histidine kinase CcaS and its cognate response regulator, CcaR, to precisely regulate the expression of superfolder green fluorescent protein (sfGFP) over time. As will be discussed below, several system features pose great challenges to this task. With our automatic experimental platform, we perform a comprehensive analysis of the performance of two different control strategies in terms of reference tracking and disturbance rejection. Using our feedback schemes, we are able to achieve highly accurate tracking and robustness to large, global perturbations to the cell culture. Finally, to demonstrate the potential of light-based feedback control in biotechnological applications as well as basic research, we use our optogenetic system to regulate the expression levels of methionine synthase—MetE—which catalyses the final, rate-limiting step of methionine biosynthesis in *E. coli* cells. By regulating MetE expression based on the continuous automatic measurements of the instantaneous culture growth rate, we present a useful and powerful application of optogenetics for long-term cell growth control.

## Results

### Experimental setup

**Figure 1:**
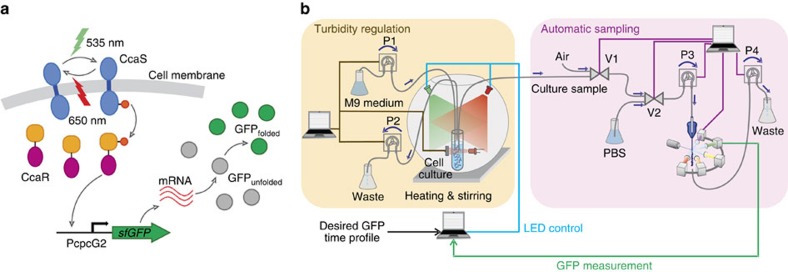
Light-switchable two-component system used in this work and experimental platform for optogenetic feedback. (**a**) On absorption of green light, the sensor histidine kinase CcaS is quickly autophosphorylated and transfers its phosphate group to the cognate response regulator CcaR. Phosphorylated (active) CcaR in turn binds to the *cpcG2* promoter to activate transcription of sfGFP. Absorption of red light inactivates CcaS, and transcription is eventually switched off. It has been hypothesized—but not yet conclusively demonstrated—that the inactive form of CcaS dephosphorylates CcaR. (**b**) Schematic of the constructed experimental platform containing the turbidity, autosampling and light-delivery modules. Straight lines denote control/measurement signals sent to/from the various devices. Curved lines indicate tubing segments. Arrows above tubing lines/pumps indicate the direction of flow/rotation. Computer icons are used to indicate control hardware and do not necessarily correspond to separate computing devices. Feedback control computations, LED control and autosampling are carried out by a single laptop, while turbidity control is coordinated by a programmable logic device. Further details are provided in the ‘Methods' section. Every 10 min., the sampling system acquires a culture sample via flow cytometry, saves and processes the sfGFP fluorescence data. On the basis of the measurement, the control algorithm determines the green-light-intensity level to be applied to the culture until the next measurement. In this way, the system can track a user-defined sfGFP expression profile.

Our experimental platform is shown on Fig. 1b. It consists of three modules: (a) an inexpensive, custom-made turbidostat to maintain constant culture conditions for arbitrarily long time spans; (b) an automatic system for sample acquisition and quantification via flow cytometry; and (c) a computer-controlled light-delivery system. All system components are controlled by Python scripts and generic microcontrollers that together enable the system to function completely autonomously. The whole platform can be easily run by a single user.

Our turbidostat follows a simple and intuitive design^18^ (see the Methods section and Supplementary Note 1). An infrared sensor measures the amount of light absorbed by the culture and feeds the measurement to a proportional-integral feedback controller implemented on a microcontroller, which computes the necessary dilution rate to maintain a given culture density. The control signal is then fed to two peristaltic pumps, one of which adds fresh medium and the other removes liquid at the same rate.

We decided to implement a custom automatic flow-cytometry setup because of the lack of low-cost commercial solutions. Our design is based on the same principles used in previous work^19^,^20^,^21^,^22^,^23^, but is tailor-made for fast sampling frequencies (up to 2 min) and minimal cross-sample contamination, disregarding more complex functions such as sample processing offered by other automatic setups. It is able to fulfil these two design goals with commonly available and inexpensive parts, such as pinch valves and peristaltic pumps. The operation of sampling setup in conjunction with the flow cytometer is coordinated from a central computer. Further details are provided in the ‘Methods' section.

Finally, our custom-built light-delivery system (see the ‘Methods' section and Supplementary Note 2) offers individual light intensity modulation for two LED groups (red and green) and contains an integrated heated magnetic stirrer that is used in conjunction with a bead bath to maintain culture temperature and aeration conditions through continuous stirring.

### Feedback control systems

The CcaS/CcaR gene expression system dynamics comprise two distinct timescales: whereas activation and deactivation are completed in a few minutes, sfGFP expression level changes can be observed on a timescale of tens of minutes, due to the slow processes of maturation and dilution. The background fluorescence and maximum induction level vary significantly from day-to-day, as does the dynamical response of the system to step changes in the light input (Supplementary Note 3). The speed of the latter also depends non-trivially on the magnitude and direction of the step^9^ (a positive-feedback mechanism arising from transcriptional read-through in the CcaS–CcaR plasmid has been suggested as a possible cause for this behaviour^24^). All these features pose great challenges to the precise dynamic control of sfGFP expression driven by the CcaS–CcaR system, emphasizing the need for closed-loop controllers.

The control algorithms implemented in this work fall under two broad categories: proportional-integral^25^ (PI) and model predictive control (MPC)^26^. Besides their very simple implementation, PI controllers guarantee zero tracking error in the steady-state for constant references, and perfect steady-state rejection of constant disturbances^25^. Moreover, PI control does not require a model of the controlled system, although a rough idea of the system response timescales is necessary for proper tuning (Supplementary Note 4). It uses the error between the current and the desired system output to compute the next control input by forming the sum of two terms: one proportional to the current error, and the other proportional to the time integral of the error (which summarizes the past error behaviour). On the downside, PI controllers are not capable of accurately tracking time-varying references, unless they change very slowly. They are also naturally better suited to systems that display a linear dynamic behaviour and require extra modifications to function adequately in the case of nonlinear systems, such as the one at hand (see the ‘Methods' section).

MPC controllers can address both of these limitations^26^. On the basis of a model of the controlled system and an estimate of its current state, an MPC controller first computes the sequence of control inputs that will bring the system output as close as possible to the reference over a given time horizon. The first step of this sequence is applied, the new system state is estimated and the whole process is repeated at the next step. Thanks to its ability to ‘look ahead', MPC can thus use the result of an optimization procedure to track complex, time-varying reference trajectories. At the same time, the iterative input computation ensures that modelling inaccuracies do not propagate in time. Therefore, tracking can be achieved with only a crude model of the controlled system. However, the improved performance comes at a computational cost, since MPC controllers are generally more complex than PI controllers (see the ‘Methods' section).

### Tracking of reference sfGFP profiles

**Figure 2:**
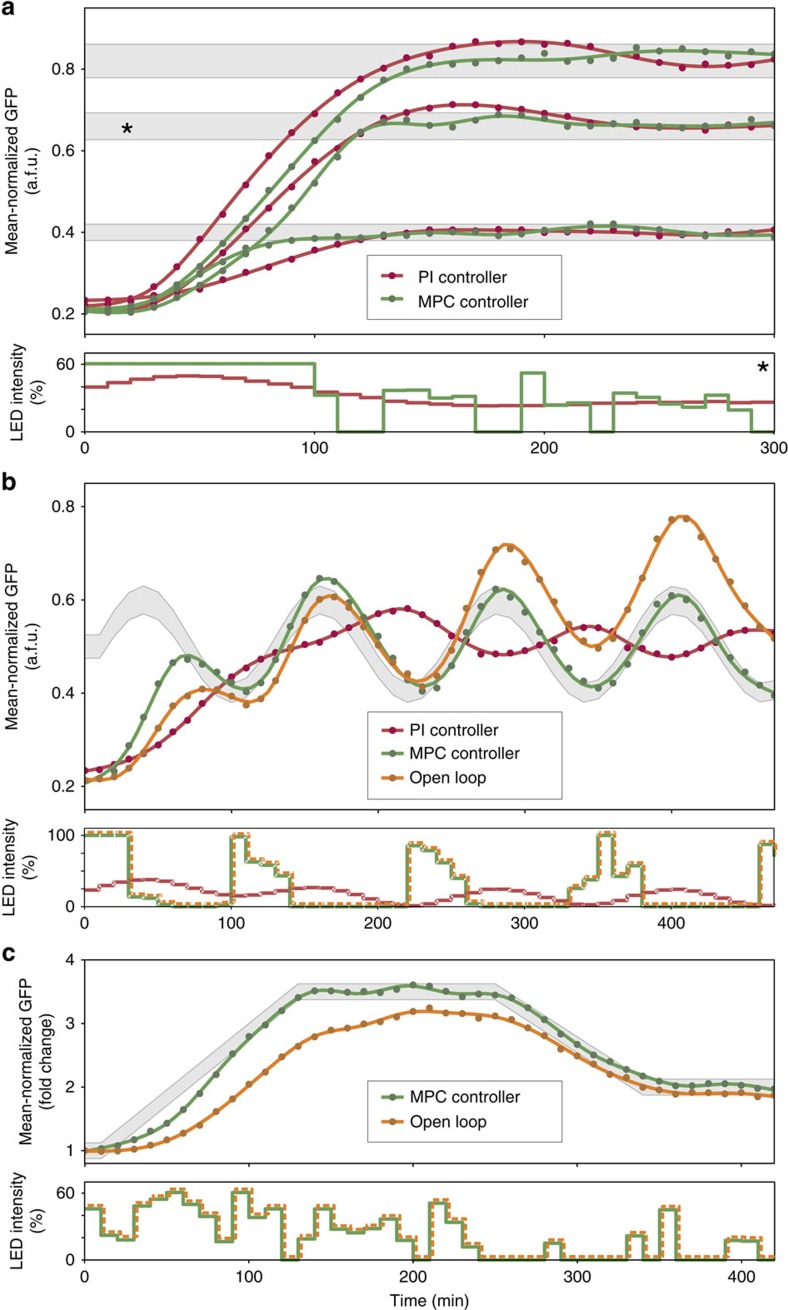
Tracking of reference sfGFP expression trajectories. Dots denote sfGFP measurements, lines are polynomial fits. (**a**) Constant reference tracking: for each constant reference level, a PI and an MPC controller were used. Grey bands determine a tolerance margin around each setpoint (±5% of the target level). It should be noted that MPC responses achieve the target (that is, stay inside the grey band) much earlier than PI responses. The applied green light inputs, expressed as a percentage of the maximum green LED intensity, are shown for the starred reference. All applied light inputs can be found in Supplementary Note 5. The maximum allowable LED intensity for MPC was limited to 60% since the dose-response curve (Supplementary Note 11) of the system is almost flat above this level. PI gains were set to *K*_*P*_=80 and *K*_*I*_=8. (**b**) Tracking of a sinusoidal reference: since the reference varies relatively fast with respect to the intrinsic timescales of the system, tracking is impossible for the PI controller (gains as in **a**). On the other hand, MPC control was still capable of excellent tracking. The grey band denotes a tolerance margin around the reference trajectory (±5% of the reference curve). To determine the repeatability of open-loop input application, the MPC input profile (green line) was applied to a culture grown on a different day, using the same growth protocol and conditions. Day-to-day variability in the dynamical behaviour of the culture is manifested in the deviation of the dark-grey response from the reference trajectory. (**c**) Tracking of a piecewise linear reference: PI control is known to generate a constant steady-state tracking error for linearly increasing inputs^25^ (unless the controlled system behaves like an integrator by itself), and was therefore not tested. MPC again achieved very good tracking (grey band depicts ±5% of the final constant level). Repetition of the MPC input profile (green line) on a different day, resulted in the dark-grey response.

Being able to achieve and maintain constant levels of protein abundance is perhaps the most basic functionality required from an optogenetic control system. We first used both PI and MPC controllers to regulate the average of normalized sfGFP fluorescence (sfGFP signal divided by forward scatter—see the ‘Methods' section for details) to several setpoints, as shown in Fig. 2a. The controllers were able to achieve and maintain the desired sfGFP levels in a reproducible manner (Supplementary Note 5) within the pre-specified tolerance levels (grey bands). Despite the careful tuning of the PI controller, MPC was able to achieve the target expression levels faster, thanks to its ability to anticipate the future behaviour of the system, which allows it to apply strong inputs at the start of the experiment to quickly increase sfGFP expression. PI controllers would need to operate under very high gain settings to achieve an equally fast rise, however, this would inevitably result in large oscillations around the target levels (Supplementary Note 4).

The PI and MPC input profiles are markedly different: PI inputs vary more smoothly over time, since the controller output cannot change too much from one measurement to the next. On the other hand MPC inputs vary more due to the fact that every input is the result of an optimization procedure that is run at every time step. Since the used model is only an approximation of the actual system, the controller is continuously revising its predictions about the future output evolution.

We next sought to investigate the tracking performance of our feedback controllers in the case of time-varying sfGFP references. Figure 2b compares the tracking performance of MPC and PI control in the case of a sinusoidal reference with a 2-h period. The advantages of MPC over PI become clear in this case: the PI control response shows a reduced amplitude and a phase shift of around 180° compared with the reference. In contrast, the MPC response stays within the tolerance margin (grey band) for the majority of the experiment. The use of real-time feedback is essential for achieving this result. To demonstrate this, we applied the same light input that was used to obtain the green curve (Fig. 2b) to a culture grown on a different day. In principle, the obtained response should be identical to the green—yet, it is not. Due to the day-to-day changes in the dynamic behaviour of the cells, and despite the fact that the cultures were prepared using the same protocol and kept under identical conditions, the dark-grey line shows a clear upward trend, and deviates significantly from the target profile.

As a final test, we used the MPC controller to track a piecewise linear sfGFP expression reference (Fig. 2c). After the initial transient, sfGFP levels remained within the tolerance band margin (±5% of the maximum reference level), while the application of the same input sequence to a culture grown on a different day again resulted in large tracking inaccuracy.

### Disturbance rejection and perfect adaptation

Disturbances are unwanted perturbations that can alter the response, the behaviour or the measured output of the controlled system, and consequently lead to gross inaccuracies in the tracking of a given reference. Disturbances in a biochemical system may arise due to unwanted interactions with its cellular environment, or changes in the external environment of the culture. In fact, complete isolation of the controlled system from its intra- and extra-cellular environment is nearly impossible. One of the principal reasons for the use of feedback control is its ability to attenuate the effect of disturbances on the system output^25^, thereby enabling robust tracking of the desired output reference. On the contrary, open-loop control is completely incapable of disturbance rejection: by their very nature, disturbances are unmodelled inputs that cannot be anticipated, and thus application of pre-computed input sequences cannot compensate for output deviations caused by disturbances.

**Figure 3:**
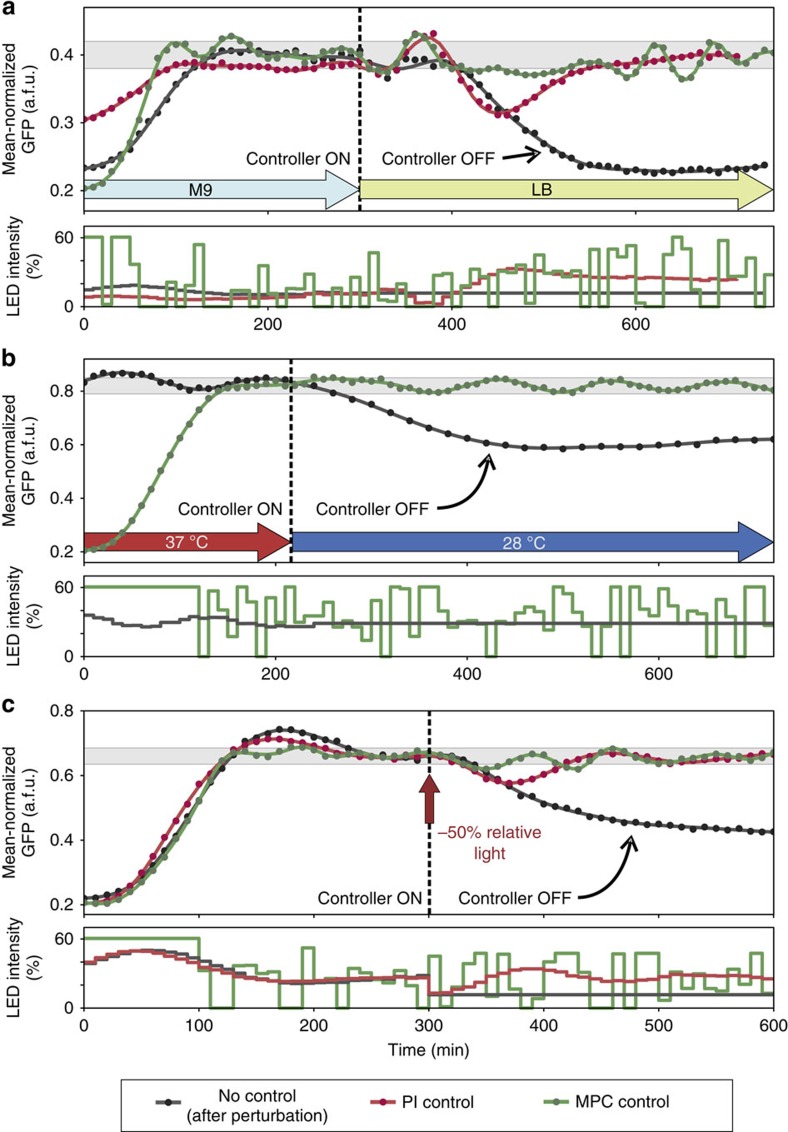
Rejection of large disturbances to the cell culture. Dots denote sfGFP measurements, lines are polynomial fits. In all panels, the grey band corresponds to ±5% of the target expression level. Each curve corresponds to a culture started on a different day. Wherever applicable, the PI gains were set to *K*_*P*_=80 and *K*_*I*_=8 from the beginning of each experiment up to an hour before the perturbation. At this point, they were changed to to *K*_*P*_=160 and *K*_*I*_=20 and the cultures were maintained for an additional hour at the same reference before the disturbance was applied. (**a**) A change of culture medium: the turbidostat feed was switched from M9 to LB at the 5 h mark, inducing a large perturbation to the culture. Day-to-day variability in controlled system dynamics led to a long transient phase for the red curve. The first part of this response is therefore not shown. For the dark-grey response we used a PI controller during the M9 phase, and switched it off after the shift, keeping the light input constant at the pre-shift level. (**b**) A decrease in culture temperature: for the dark-grey response we used a PI controller during the 37 °C phase to achieve the desired sfGFP fluorescence. The transient phase of the PI response is not shown on the plot. The PI controller was switched off after the cells were taken out of the heat bath and light input was kept constant at the pre-shift level. (**c**) An input perturbation: after 5 h into the experiment and until the end, the green light input was reduced by subtracting 50% of the PI input at 5 h, to mimic a damage in the light-delivery system. For the dark-grey response we used a PI controller during the first 5 h, and switched it off after the perturbation, keeping the light input constant at the reduced level.

To demonstrate the disturbance-rejection capabilities of our feedback controllers, we performed a series of large, global perturbations to our cell cultures (Fig. 3). For every test, the average of normalized sfGFP (see the ‘Methods' section) was first driven to a pre-specified reference level using feedback control. The cultures were then perturbed, and the controllers were left to automatically compensate the effect of the perturbation and bring the output back to the target reference. The performance of controlled cultures was compared with the behaviour of cultures in which the PI feedback controller was turned off at the time of perturbation, and its output was maintained at a constant level equal to its value just before the perturbation. The use of PI control for these cultures during the pre-perturbation phase was motivated by the fact that this controller can ‘learn' the constant light input required to maintain the system at the desired reference level, whereas the MPC input fluctuates in time.

In our first test, the turbidostat feed of cultures grown in M9 minimal medium was abruptly switched to richer LB medium. This resulted in a marked change in cellular growth and morphology: within an hour after the perturbation, cell size increased significantly (Supplementary Note 6), while the culture-doubling time shifted from ∼38 min in M9 to ∼25 min in LB. These large-scale changes reflect the massive re-organization of cellular metabolism that takes place in the shift from a minimal to a rich growth medium. Its effect can be seen in the complete collapse of the uncontrolled system output (dark-grey line) on Fig. 3a. In sharp contrast, both MPC and PI controllers manage to bring the output within the tolerance band (±5% of the target).

For the second test, cultures grown at 37 °C were abruptly shifted out of the heat bath and left to cool down to 28 °C over the course of 30 min. (Fig. 3b). Consequently, the doubling time increased from ∼38 min to >1 h, while cells shrunk in size (Supplementary Note 6). While the fluorescence of the uncontrolled culture eventually decreased slowly over time, the MPC controller was able to maintain the system within the tolerance band (±5% of the target) at all times after the perturbation. The use of a PI controller in this case would be problematic: as the system becomes increasingly unresponsive at 28 °C and slows down considerably, the accumulation of large integral errors in the PI controller would lead to highly oscillatory outputs unless an anti-windup^27^ scheme was used.

In the final test, an artificial input perturbation was introduced to the system by subtracting 50% of the LED intensity that was applied by the PI controller 5 h into the experiment, after steady-state had been achieved (Fig. 3c). In this case, the cells received a smaller amount of green light after the perturbation, and the feedback controllers had to compensate for the decrease by requesting the application of larger control inputs. The dark-grey line demonstrates the effect of the input reduction, as the cells shift to a lower steady-state fluorescence. On the other hand, both controllers are able to reject the disturbance and move the system back to the tolerance band (±5% of the target).

### Optogenetic control of cell growth

The powerful capabilities of optogenetic regulation can be very advantageous for the control of biologically relevant cellular processes, such as cell growth. One significant challenge in bioprocess regulation is the control of biomass accumulation, with the aim of optimizing the production efficiency of desired chemicals or proteins while minimizing the accumulation of toxic byproducts. A classical approach for limiting growth is the use of rifampicin to inhibit the bacterial RNA polymerase, and thus increase T7 RNA polymerase-driven recombinant protein expression^28^. Others include the limitation of a growth substrate, typically glucose^29^ or phosphate^30^, and regulation of the growth rate with temperature^31^. The first two approaches suffer from significant drawbacks: antibiotics may cause cell death and are costly; on the other hand, nutrient limitations affect both catabolism and anabolism, leading to global metabolic changes that may adversely affect the accumulation of the desired product. Finally, the maximal speed of temperature changes is limited by equipment capabilities and the culture volume.

In recent work, a synthetic growth switch in *E. coli* based on the inducible expression of the β and β′ subunits of RNA polymerase was presented^32^. That system displays an ultrasensitive response of growth rate to subunit expression, while it relies on IPTG for induction. For increased flexibility, a better titratable system with easily reversible induction would thus be desirable. Following an alternative path, we chose to control protein synthesis and, ultimately, cell growth by modulating the expression levels of the *E. coli* methionine synthase—MetE.

*E. coli* has two methionine synthases, the cobalamin (B_12_)-dependent MetH and B_12_-independent MetE synthase. Since *E. coli* is incapable of synthesizing B_12_, MetE is the only functional enzyme that catalyses *de novo* methionine synthesis in the absence of exogenous B_12_ (ref. 33). Furthermore, MetH abundance appears to be much smaller than MetE in minimal medium^34^. MetE is thus essential for methionine synthesis in a methionine-dropout medium, and is therefore greatly upregulated under these conditions^34^. Being a slow enzyme, MetE also catalyses the rate-limiting step in methionine biosynthesis^33^,^34^. Due to the fact that *N*-formylmethionine is required to initiate translation of all proteins, changes in MetE abundance should have an immediate impact on methionine availability and, ultimately, protein synthesis. As was recently experimentally verified^24^,^35^, MetE levels indeed determine the cell growth rate.

**Figure 4:**
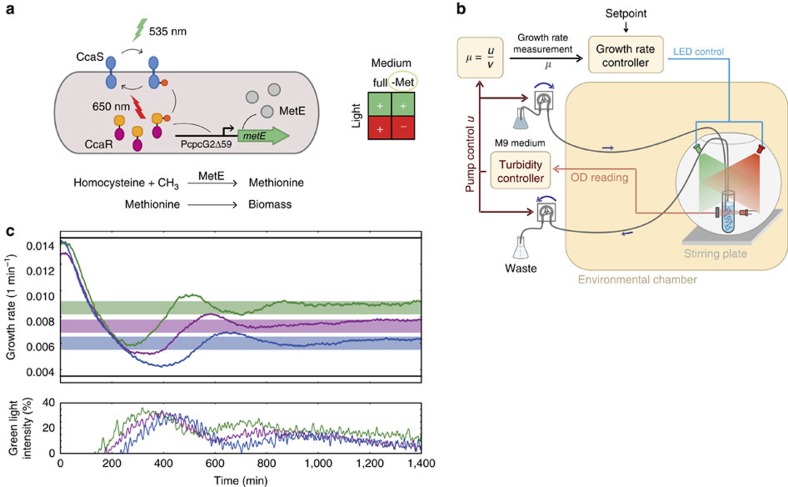
Light-inducible cell growth control. (**a**) MetE catalyses the final step in *de novo* methionine synthesis by converting homocysteine to methionine through the addition of a methyl group^33^. Deletion of the endogenous *metE* and placement of a chromosomally integrated copy under the control of a modified *cpcG*2 promoter (*P*_*cpcG2Δ59*_) that is activated by CcaR results in light-inducible growth-rate modulation when cells are grown in M9 medium lacking methionine. (**b**) Nested feedback loops for turbidity and growth-rate regulation. The inner turbidity control loop maintains a constant culture optical density as described above. The outer control loop monitors the influx pump control signal, *u*, to infer the culture growth rate after some simple signal-processing operations (see the ‘Methods' section and Supplementary Note 7). A PI controller (see the ‘Methods' section) makes use of this information and modulates the ratio of green-to-red intensities so that the culture achieves a user-defined growth rate within the dynamic range of the system. (**c**) Coloured lines (upper panel): tracking of constant growth-rate setpoints using a PI controller (PI gains: *K*_*I*_=45 for all curves, and *K*_*p*_=6,000, 8,000 and 10,000 for the green, magenta and blue curves, respectively). The applied light inputs (lower panel), expressed as a percentage of the maximum green LED intensity (see the ‘Methods' section), are colour-coded according to the corresponding growth-rate curve. Solid black lines: minimum and maximum achievable growth rates. Under full-intensity green light, the cells achieve a growth rate of around 0.0139, min^−1^ (doubling time of ∼50 min). Growth under full-intensity red light reduces the growth rate to about 0.0035, min^−1^ (doubling time of ∼200 min), but it should be noted that after prolonged growth under red light, the cells start to adapt and grow at a faster rate.

An open-loop control scheme for MetE had been suggested in the literature^24^, and we therefore first sought to reconstruct the strain used in that study. Unfortunately, with our experimental setup and growth protocols, the cells failed to grow without methionine, regardless of the light conditions. This is presumably due to several limitations of the original CcaS/CcaR system, outlined in the ‘Methods' section. To affect the growth rate of a liquid culture grown in methionine-dropout minimal medium, we instead coupled a refactored version of the CcaS/CcaR system featuring reduced transcriptional leakage and higher dynamic range (see the ‘Methods' section) with the methionine biosynthesis pathway. This was achieved by integrating a single copy of *metE* under CcaR-dependent transcriptional control into the chromosome of a Δ*metE* strain (Fig. 4a and see the ‘Methods' section). The deletion strain with light-inducible MetE expression had a doubling time of around 55 min when grown under full green light, which is a bit longer than the 38 min doubling time of the sfGFP strain described above. On the other hand, growth under red light increased the doubling time to about 200 min (Fig. 4c and Supplementary Note 7).

To measure the instantaneous growth rate, we made use of the fact that when culture density is maintained constant inside the turbidostat, the supply rate of the influx pump is directly proportional to the growth rate (Fig. 4b and see the ‘Methods' section). In this way, after proper conversion of the turbidostat controller output into flow rate and the necessary signal-processing steps (Supplementary Note 7), the growth rate can be sampled at a high-frequency rate (∼1 Hz) and fed into an external PI controller that tracks a user-provided target growth rate by appropriately modulating the green-to-red light intensity ratio (Fig. 4b). The final closed-loop system thus consists of an inner control loop for constant cell-density maintenance, and an outer control loop for light-based growth-rate control (Fig. 4b). In this scheme, the output of the inner loop controller (the inflow rate of fresh medium) is used as the input to the growth-rate controller in the outer loop.

Contrary to the relatively well-understood dynamics of the GFP expression system, regulation of MetE generates a feedback loop between global cell physiology and gene expression, whose dynamics is much harder to describe. Despite this complexity, a PI controller proved to be sufficient for precise growth-rate regulation, thanks to the slow dynamics of the overall system. As the results of our tracking experiments demonstrate (Fig. 4c and Supplementary Note 7), the controller was able to achieve and maintain a desired growth rate within the available dynamic range, despite a drift in the light sensitivity of the cells over time resulting from undesired selection effects (see the ‘Methods' section). This can be observed in the small downward trend of the green light-intensity curves of Fig. 4c and the data of Supplementary Note 7. This drift is a consequence of the PI controller adaptation to the gradual speed up of the cells—essentially a disturbance-rejection property that no open-loop control scheme could achieve.

## Discussion

We have presented an integrated framework for automatic optogenetic feedback of liquid cell cultures that comprises tailor-made hardware and software. With its help, we have been able to achieve excellent precision in the regulation of protein expression driven by a light-switchable two-component system in *E. coli*. Moreover, we have shown how feedback operation enables the system to function reliably even in the presence of large global perturbations to the culture, such as a change of the growth medium, a temperature shift or an input perturbation. Taken together, these benefits demonstrate the advantages of feedback regulation over open-loop control approaches typically reported in the optogenetics literature.

Our experimental platform also enabled us to explore the accuracy/complexity trade-offs of two different feedback-control schemes, and investigate their interplay with the biological system under study. Our results on the control of continuous cell cultures nicely complement recently presented work^36^, which compared the performance of three alternative control strategies (PI, MPC and Zero Average Dynamics) for the regulation of fluorescent protein levels using a galactose-inducible system in yeast grown inside a microfluidic device. They also constitute a significant extension, both in terms of complexity and accuracy, of the work presented in ref. 37.

Besides the feedback-control algorithms tested in this work, our experimental platform can be easily used with alternative, possibly more advanced, controllers and serves as a test bed for various control approaches from the rich automatic control literature^38^.

Apart from biotechnology applications, our platform also naturally lends itself to the generation of controlled perturbations for the characterization of endogenous intracellular pathways^2^, as well as the closed-loop identification of cellular networks^39^ and the design of optimal control inputs for parameter inference^40^ and/or model selection.

Our system can be expanded in several directions: the use of parallel continuous cell cultures and multiplexed sampling (for example, with the help of a simple *x*-*y*-*z* robotic arm) will accelerate data collection and speed up the controller-tuning process. On the other hand, more complex feedback-control tasks (such as the simultaneous control of protein mean and variance over a cell population) can be accomplished via the incorporation of multiple orthogonal optogenetic systems within the same cell^41^, and the use of multivariable control techniques^42^.

To demonstrate the capabilities and potential of optogenetic feedback, we chose to dynamically regulate the culture growth rate by placing the supply of intracellular methionine under optogenetic control, thus effectively controlling the global protein-synthesis rate in the cells. Despite the increased dynamical complexity of the resulting system, we demonstrated that precise growth-rate regulation is possible and indeed achievable without the use of sophisticated measurement systems, simply by monitoring a control signal in our turbidostat. Furthermore, the ability to control the growth rate can be also be used to study how bacterial physiology responds to dynamic variations in global protein-synthesis rates^43^, thus overcoming the limitations of studies focusing on steady-state growth.

Ultimately, the measurement automation and light-control systems, as well as the control algorithms used here could be used for online monitoring and optogenetic feedback control of cells inside bioreactors. In the field of bioprocess regulation, external feedback using appropriate biosensors and inducers has already been proposed as a means to improve productivity, robustness and batch-to-batch reproducibility^14^. Light would be an ideal inducer for gene-expression control of these systems, as it can provide targeted, non-toxic and bidirectional regulation, which more-expensive chemical inducers cannot achieve. On the other hand, there are great challenges that need to be addressed for this approach to be technically and economically viable. Bioreactors typically operate at very high cell densities and are opaque. Delivering light to large-volume, dense cell cultures will thus require the careful study, design and construction of the light sources for this task, taking into account issues such as mechanical stresses, electrical wiring and mixing efficiency. The use of optogenetic induction systems optimized for maximum sensitivity and slow dark reversion^44^,^45^, implies that very low-intensity (or even intermittent^46^) exposures will be sufficient for induction and may help in this direction. Recent experimental results on the open-loop optogenetic regulation of gene expression in small mammalian cell bioreactors^47^ make us optimistic that case-specific solutions will be feasible in the future. Regardless, optogenetics may eventually be very useful in fast prototyping of new metabolic pathways or tuning existing metabolic networks in small-scale lab setups.

## Methods

### Strains and plasmid construction

*E. coli* strains, plasmids and primer sequences used in this study are listed in Supplementary Table 1 (Supplementary Methods). Strain JT2 containing the phycocyanobilin (PCB) biosynthesis plasmid pPLPCB(S) and *ccaS*/*ccaR* plasmid pJT119b was used for all fluorescence-based feedback-control experiments^9^. Strain JT2 Δ*metE::FRT Tn7::FRT-PcpcG2Δ59-metE*-containing plasmids pPLPCB(S) and pSKA413 was used for the cell-growth-control experiments (details provided in Supplementary Methods). Plasmids, plasmid sequences and the host strain constructed for cell-growth-control experiments are available at Addgene (#80380, #80381, #80403).

### Culture media

Cells were grown in LB (1% tryptone, 0.5% yeast extract, 1% NaCl) or M9 medium supplemented with 0.2% casamino acids, 0.4% glucose and 0.001% thiamine unless otherwise indicated. For the growth-control experiments, M9 methionine-dropout medium supplemented with 0.4% glucose, 0.001% thiamine and 19 amino acids (40 μg ml^−1^) was used. Antibiotics were used at the following concentrations: chloramphenicol, 34 μg ml^−1^; spectinomycin, 100 μg ml^−1^; ampicillin, 100 μg ml^−1^; kanamycin, 40 μg ml^−1^.

### Growth conditions

All overnight and turbidostat cultures, with the exception of the culture shifted to LB, were grown in M9 medium.

*Overnight growth and initialization protocol for GFP experiments.*

1. Two 5 ml cultures are started from the −80 °C glycerol stock on the evening before the experiment. Cells are grown in black 15 ml centrifuge tubes (TB1500, Argos Technologies) containing M9 medium with antibiotics at 37 °C with shaking at 230 r.p.m. Since CcaS is produced in its inactive form, the background sfGFP fluorescence of cells grown in the dark is the same as that of cells grown under red (inactivating) light. The starting OD_600_ is set in such a way that the cells grow in exponential phase (after a ∼1 h lag) for 10–12 h before the beginning of the experiment the next morning, and are at OD_600_ 0.08–0.1 right before being transferred to the light-delivery system.
2. The next morning, after an OD_600_ measurement to verify overnight growth, the culture is transferred into a 25 ml glass centrifuge tube (Schott 2160114, Duran) with a 3 × 8 mm magnetic stir bar (13.1120.02, Huberlab) that is placed inside the bead bath of the light-delivery system. M9 medium (w/antibiotics) is added to a final volume of ∼17–18 ml. The cells are left to settle under red light illumination at 37 °C with stirring at 1,500 r.p.m. while the sampling system and turbidostat are set up.

*Overnight growth and initialization protocol for growth control experiments.*

1. Overnight cultures are grown in M9 medium inside clear culture tubes and green light illumination, to stimulate the production of MetE. The starting OD_600_ is set in such a way that the cells are still in exponential phase (OD_600_<0.2) on the following morning.
2. The next morning, the overnight cultures are centrifuged and washed twice with M9-dropout medium lacking methionine before being transferred into 25 ml glass tubes containing M9 methionine-dropout medium. Before the start of the growth-control experiments, the cells are grown inside the light-delivery system (37 °C with stirring at 1,500 r.p.m.) under full-intensity green light for ∼6–7 h, to reach their maximal steady-state growth rate in the dropout medium.

### Automatic sampling setup

The automatic sampling setup (Fig. 1b) is composed of two Verderflex EZ OEM peristaltic pump heads mounted on custom-made housing that contains the driving circuits (in-house); two three-way solenoid pinch valves (PS-1615W 12VDC, Takasago) powered by a driving circuit (in-house); and segments of silicone tubing (1.6 mm Φ_internal_ × 3.2 mm Φ_external_, Cole Parmer) joined together with wye connectors (1/16′′; 5463K51, McMaster-Carr). The pumps and the valves are controlled by an Arduino UNO microcontroller, which is itself controlled by custom-written Python code.

Each culture sample is ∼80 μl. To deliver the sample to the cytometer, we use pump P3 (Fig. 1b). The sample is first isolated by air and then pushed across the silicon tubing using phosphate-buffered saline (PBS, Sigma-Aldrich), since the pumps work more reliably when pumping liquid. More concretely, the system goes through the following sequence of steps:

1. start pumping of liquid culture sample,
2. once a small amount of the sample is beyond valve V1 (Fig. 1b), switch V1 to air (sample isolation),
3. once air is past V2, switch V2 to PBS and push the air+culture sample to the cytometer,
4. add air behind PBS once the sample reaches the cytometer (isolation from the next sample). Note, after the above operations the isolated culture sample and some of the PBS behind it (used for dilution) have reached a polystyrene sampling tube (ST; 55.484.001, Sarstedt) that is placed under the cytometer sample introduction port,
5. pump air into the tube segment behind V1 and
6. push back remaining sample into the culture.

While pump P3 is working, pump P4 removes the PBS that ends up in ST and brings it to a waste flask. In this way, both the tubing and the ST are cleaned from the previous sample. After the current sample is placed under the sample introduction port, the cytometer software (BD Accuri C6 CFlow Software 1.0.264.15) is operated via a custom automatic clicking program written in Python. The following steps are performed by the clicking script:

1. sample acquisition,
2. data storage and export into FCS format and
3. cytometer backflush.

After measurement is completed, pump P4 removes all liquid from the ST. Due to the fact that the used cytometer (BD Accuri C6) is operated by proprietary software that cannot be manipulated externally, the use of the clicking script provides a very simple and flexible solution to cytometer control, and is easily configurable for any cytometer.

With the measurement protocol outlined above, cross-contamination between consecutive samples is minimal, and data obtained from automatic measurements are indistinguishable from the data obtained by manual sampling (Supplementary Note 8). Measurements were obtained every 10 min.

### Turbidity control

Cell density during experiments was maintained at an OD_600_ of 0.1 via a turbidostat composed of two peristaltic pumps (Preciflow, Lambda-instruments) with an RS-232 interface that is controlled via a microcontroller. A photodiode (BP104, Vishay Semiconductors) and an infrared LED (LD274-3, Osram Semiconductors) are used to measure culture turbidity. The infrared LED has a peak emission wavelength of 950 nm, which is well-separated from the longest wavelength at which the red-absorbing form of CcaS shows any absorption (∼750 nm) (ref. 48). The photodiode measurements are sent to a PI controller implemented in the microcontroller, which maintains turbidity at a pre-specified value by operating the peristaltic pumps. A programmable logic controller was used as the microcontroller for the GFP experiments. It was replaced by an Arduino for the growth-control experiments, because of its amenability to being interfaced with a computer. Media is fed and removed from the culture via silicone tubing (1.6 mm Φ_internal_ × 3.2 mm Φ_external_, Cole Parmer). Additional details are provided in Supplementary Note 1.

### Light-delivery and heating/stirring system

The light-delivery system consists of a lightproof spherical metal shell (constructed from common household materials), on which the green (523 nm) and red (660 nm) LEDs (LZ1-30R200 and LZ1-00G100, LEDengin) and their heat sinks are retrofitted. LED intensity is controlled by an LED driver (in-house) using high-frequency pulse-width modulation. The system is integrated with a heated magnetic stirrer (VMS-A, VWR) for heating and stirring. The culture tube is placed inside a 1,000 ml glass beaker that contains hot metal beads (Lab Armor), which in turn is placed on the heating surface. The bead temperature is kept at 37 °C (with the exception of the temperature-shift experiment).

For the growth-control experiments, the heating plate was switched for an incubation hood (Sartorius Certomat HK), as the latter proved to be more precise. The improved temperature control is required because the readout of the turbidity sensor is sensitive to small temperature variations.

### Light-treatment protocol

The activity of the CcaS–CcaR system is controlled by the ratio of green-to-red light intensity under continuous illumination conditions^9^,^17^. We therefore used for all our GFP control experiments a constant red light illumination, determined as the maximal red intensity at which operation of the green LEDs at full power still results in the maximal fold change in mean sfGFP fluorescence. For growth-rate control the green intensity was determined by the controller, while the sum of green and red LED intensities, normalized to their respective maxima, was kept constant; in this way, a change in green intensity is reflected in an opposite change in red intensity.

Green light intensity was kept constant at values determined by the control algorithms between measurement times (10 min for GFP experiments and 1 min for growth-control experiments) and was always expressed as a percentage of the maximum green LED intensity. The duty cycle of the pulse-width modulated LED current was used as a proxy for LED intensity. These quantities follow a perfectly linear relationship, as we verified using a light-to-voltage sensor (TSL 235T, TAOS).

### Flow cytometry

Cells were measured on a BD Accuri C6 flow cytometer (BD Biosciences) using an Argon ion laser (488 nm) and FL1 filter (530/30 nm). During the acquisition phase, between 1,000–2,000 events per second were measured with a 14 μl per min flow rate. An FSC-H threshold of 11,000 a.u. (typical cell mean is >30,000 a.u.) was used to remove events due to instrument noise. Every sample contained 20,000 events collected inside a very wide elliptical gate in the FSC-A versus SSC-A space (enclosing the whole cluster corresponding to cellular events), to further eliminate events due to instrument noise. Raw cytometry data are processed with custom Python scripts as follows: events with fluorescence below a threshold of 800 a.f.u. (cells have a mean of ∼6,000 a.f.u.) or outside an elliptical gate on the FSC-A versus SSC-A scatter plot are discarded. The ellipse is described by the inequality


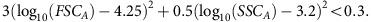


After gating, ∼15,000 events are left. Fluorescence values are then normalized with respect to FSC-H, which is known to be positively correlated with cell volume^49^. We thus obtain a quantity that reflects the concentration of sfGFP within each cell. The sample mean of the normalized single-cell sfGFP measurements is used to represent the culture fluorescence at the time of measurement. Further details are provided in Supplementary Note 9.

### Description of control algorithms

Both control algorithms are allowed to change the system input (green light intensity) only when a new output measurement is available. Between measurement instants, the input to the controlled system remains constant. This is the simplest instance of so-called digital control^25^.

*PI control*. Denoting the actual system output at measurement time *t*_*k*_ by *y*(*t*_*k*_) and the desired (reference) output at the same time by *y*_*ref*_(*t*_*k*_), the error *e*(*t*_*k*_)=*y*_*ref*_(*t*_*k*_) −*y*(*t*_*k*_) is formed. The PI controller contains two terms: one proportional to the current error value (called the proportional term) and one that takes into account the past values of the error, formed by the sum of error values from the beginning of the experiment until the current time (called the integral term). Thanks to the integral term, PI controllers can achieve zero steady-state tracking error for constant (or slowly varying) references, as well as perfect rejection of constant disturbances. Mathematically, the input applied to the system in the GFP control experiments is given by


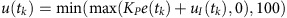


where


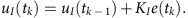


The applied input, *u*(*t*_*k*_), is expressed as a percentage of the maximal green LED intensity, and is saturated within the physically reasonable interval [0,100]. At the beginning of the experiment (time *t*_0_), the integral term is initialized to *u*_*I*_(*t*_0_)=0. The controller contains two free parameters (also called gains), whose choice affects the behaviour of the *closed-loop* system (that is, the system formed by the feedback interconnection of the controlled system and the controller).

As we noted above, the dynamics of the CcaS–CcaR system feature nonlinearities that couple the speed of the transcriptional response to the magnitude of input changes^9^. Practically, this means that the system becomes almost unresponsive to small changes in the light input. Therefore, a PI controller tuning that can bring the output to the desired setpoint is not adequate for fast disturbance rejection, which typically requires larger sensitivity to output deviations (Supplementary Note 10). To overcome this limitation, we applied a so-called gain scheduling approach, by using a first set of PI gains to bring the system to the desired setpoint, and then switching to a set of larger gains to increase the controller sensitivity to small output deviations.

For the growth-control experiments, the PI control loop is updated every minute with a filtered growth-rate measurement *μ*(*t*_*k*_). The proportional and integral terms are then obtained as follows:


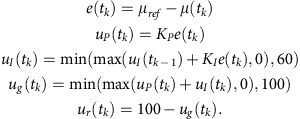


In the above equations, *μ*_*ref*_ is the target growth rate, *e*(*t*_*k*_) the corresponding error. 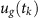
is the green light input that will be applied to the culture during the next minute, and 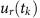
is the corresponding value for the red light. Both *u*_*r*_ and *u*_*g*_ are expressed as percentage of maximal LED intensity. Finally, the values for *u*_*I*_(*t*_*k*_) are saturated within the interval [0, 60], to prevent the accumulation of too large positive or negative errors in the integral term and improve the dynamic performance of the controller. The controller gains, *K*_*P*_ and *K*_*I*_ were chosen by manual tuning.

*Model predictive control*. MPC makes control input decisions based on a dynamical model of the controlled system. We therefore constructed a model whose states correspond to the means of sfGFP mRNA (*r*), immature sfGFP (*p*) and fluorescent sfGFP (*g*). Their dynamical evolution is described by a system of three coupled linear ordinary differential equations:


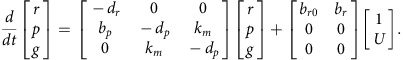


According to these equations, sfGFP mRNA is produced at a rate proportional to the applied input (green intensity), *U*, and degraded with rate *d*_*r*_. Immature sfGFP is produced from mRNA with rate *b*_*p*_, diluted with rate *d*_*p*_ and converted to fluorescent sfGFP with rate *k*_*m*_. Even when *U*=0 (that is, cells are grown under purely red light or in the dark), the background kinase activity of CcaS results in a non-negligible amount of phosphorylated CcaR, and hence a basal transcription rate *b*_*r*0_.

To arrive at the final MPC model, the above system was converted to a fold-change model, which describes the changes of the species over their initial conditions in the un-induced system (*U*=0). In the fold-change coordinates (denoted with capital letters), the system equations are


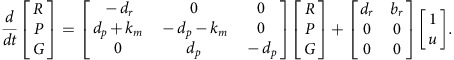


Assuming that the system is at the un-induced steady-state (*u*=0) at *t*=0, the initial condition of this system is *R*(0), *P*(0), *G*(0)=1. With this change in state coordinates, the new input (now denoted by *u*) expresses the additional steady-state sfGFP fold change that a given green intensity, *U*, can achieve. For example, *u*=1 will lead at a steady-state *R*_*ss*_, *P*_*ss*_, *G*_*ss*_=2. The new input is related to the green light intensity through the dose-response curve of the system (the relation between constantly applied green intensity and steady-state GFP). That is, *u*=*f*(*U*), with *f* estimated from a set of steady-state sfGFP measurements at various green-light-intensity levels (Supplementary Note 11). A maximum-likelihood fit of the above model to a characterization data set consisting of a step response together with a few PI control responses, gave the nominal parameter values *d*_*r*_=0.0956, *d*_*p*_=0.0214, *b*_*r*_=0.0965 and *k*_*m*_=0.0116.

This continuous-time model was then converted to an equivalent discrete-time system^25^ with sampling rate *T*_*s*_=10 min. Assuming that some disturbances can be modelled as uncontrolled inputs to the system, we appended an extra input variable, *d*(*t*), to the model^50^. In the undisturbed (nominal) system, *d* remains constantly at zero. The value of *d* is estimated at every measurement, to assess whether the nominal system has been perturbed. Finally, a time-delay equal to one sampling period (10 min.) was added to the system input, to reflect the fact that inputs applied at time *t* can actually affect the system at time *t*+*T*_*s*_ (presumably due to the unmodeled CcaS–CcaR dynamics). In vector form, the discrete-time model equations are then


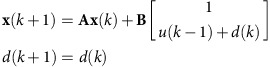


where, *k* denotes the number of time steps (that is, multiples of *T*_*s*_) elapsed since the beginning of the experiment, 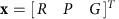 and 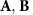 are matrices that depend on the system parameters. Further details are provided in Supplementary Note 12.

At every measurement time, only the last model state (*G*) is measured. However, to predict the future evolution of the system, the MPC algorithm requires knowledge of all system variables (*R*, *P*, *G*). Therefore, they need to be estimated whenever the value of *G* is updated.

Moreover, not all disturbances can be assumed to enter the dynamics additively, as *d* does. For example, a change in the cell-doubling time has to be reflected in a change of *d*_*p*_. Since MPC is model-based control, its tracking performance largely depends on the accuracy of the model it uses. A model calibrated for growth in M9 medium at 37 °C will inevitably lead to unacceptable controller performance when the medium or temperature are changed. This prompted us to implement an adaptive scheme, where all the system parameters (*d*_*r*_, *d*_*p*_, *b*_*r*_, *k*_*m*_ and the disturbance *d*) are also estimated at every measurement, to detect changes in the dynamic behaviour of the system.

Joint state and parameter estimation was performed using a powerful recursive Bayesian estimation, called particle filtering. The description of the particle filter^51^ used in this work is given in Supplementary Note 13.

Our MPC+particle filtering schemes, presented in detail in the Supplementary Notes 13 and 14, were implemented in Matlab scripts and were evoked by the master Python script that controlled the execution of each experiment. Despite its apparent complexity, computation of a light input was performed within 20 s from the moment of measurement acquisition, using our speed-optimized scripts.

### Light-inducible control of MetE

The pJT119b plasmid used in sfGFP control experiments has some severe limitations that make it inapplicable in the growth-control study. As previously reported^24^, the abundance of the sensor kinase CcaS does not remain constant under red and green illumination, most likely due to transcriptional read-through from the upstream *sfGFP* gene in pJT119b. Besides coupling CcaS and GFP expression, the read-through adds unnecessary complexity to the dynamics of the light-induction system, which ideally should be kept as simple as possible.

Another limitation of the original pJT119b plasmid is the relatively high transcriptional leakage observed in red light, which has been attributed to the presence of a constitutive promoter sequence embedded in the *cpcG*2 promoter driving sfGFP^52^. By elevating the background expression levels of the system, this leakiness reduces the dynamic range of the system and limits it usability for control of downstream gene expression.

To overcome these limitations, we constructed plasmid pSKA413 (Supplementary Methods) by refactoring the pJT119b plasmid as follows: C-terminally FLAG-tagged *ccaS* was transcriptionally fused downstream of a C-terminally FLAG-tagged *ccaR* and a T7 transcriptional terminator was added. In all, 59 base pairs corresponding to a putative constitutive promoter within the *cpcG2* promoter region^52^ were deleted (*P*_*cpcG2Δ59*_), and the *sfgfp* reporter was exchanged for a *gfpmut3* reporter with a synthetic RBS designed using the RBS Library Calculator^53^.

Plasmid pSKA413 and PCB biosynthesis plasmid pPLPCB(S)^54^ were introduced into a modified strain JT2 deleted for endogenous *metE* and containing a chromosomal Tn7 insertion of *metE* under control of the *cpcG2Δ59* promoter.

The resulting light-induction system displays an order of magnitude greater dynamic range in comparison with pJT119b (Supplementary Fig. 3 in Supplementary Methods), while the expression of the sensor kinase is independent of the light conditions. A refactored and optimized version of the CcaS–CcaR system was also presented in ref. 52, which also featured substantially better induction range and lower transcriptional leakage. In ref. 52 *ccaS* and *ccaR* are located on different plasmids: *ccaR* lies together with the *sfgfp* gene on one plasmid, while *ccaS* is transferred to the PCB expression plasmid. Both genes are driven by synthetic promoters and ribosomal-binding sites. In contrast, in our plasmid *ccaS* and *ccaR* are coexpressed in *cis* under the endogenous *ccaR* promoter, with their individual expression levels being independently adjustable through the use of synthetic ribosomal-binding sites. This results in a more compact construct and decreased variability in CcaS and CcaR protein abundance due to varying plasmid copy numbers. A basic characterization of our refactored system is presented in Supplementary Methods.

*Phenotypic stability of the strain*. After prolonged growth under growth-repressive conditions (24–48 h), the strains appear to gradually lose sensitivity to light and speed up, most likely due to selection effects stemming from the fact that they still harbour the CcaS/CcaR system on a medium-copy plasmid.

### Growth-rate estimation in the turbidostat

To see how the culture growth rate can be inferred from the influx pump control signal, *u*, consider the equation for the accumulation of biomass,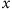, in non-limiting substrate conditions^55^: 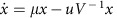, where *μ* is the growth rate, *u* the medium inflow rate (ml min^−1^) and *V* is the culture volume. When *x* is maintained constant, the growth rate is thus equal to the dilution rate, *uV*^−1^. The doubling time, *T*_*d*_, can be computed via 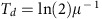.

Under dynamically changing light inputs, the culture growth rate varies in time. Since growth rate evolves on a timescale of hours, it is much slower than the establishment of equilibrium inside the turbidostat culture tube. The speed of the latter is determined by the tuning of the turbidity-regulating PI controller and ranges from a few seconds to a few minutes. Biomass concentration is therefore maintained constant by the turbidity loop despite the changes in cell growth, and the instantaneous growth rate can still be estimated as shown above.

### Data availability

All relevant data (design schematics, computer code and experimental data) are available from the authors on request. Cell growth-control plasmids and host strain are available at Addgene (#80380, #80381 and #80403).

## Additional information

**How to cite this article:** Milias-Argeitis, A. *et al*. Automated optogenetic feedback control for precise and robust regulation of gene expression and cell growth. *Nat. Commun.* 7:12546 doi: 10.1038/ncomms12546 (2016).

## Supplementary Material

Supplementary Information Supplementary Figures 1-4, Supplementary Table 1, Supplementary Notes 1-14, Supplementary Methods, Supplementary References.
