# Supplementary material for: Automated optogenetic feedback control for precise and robust regulation of gene expression and cell growth
Source: Nat Commun. 2016 Aug 26;7:12546. doi: 10.1038/ncomms12546 (PMC5007438; doi:10.1038/ncomms12546)
Supplement: Supplementary Information — Supplementary Figures 1-4, Supplementary Table 1, Supplementary Notes 1-14, Supplementary Methods, Supplementary References. [file ncomms12546-s1.pdf]

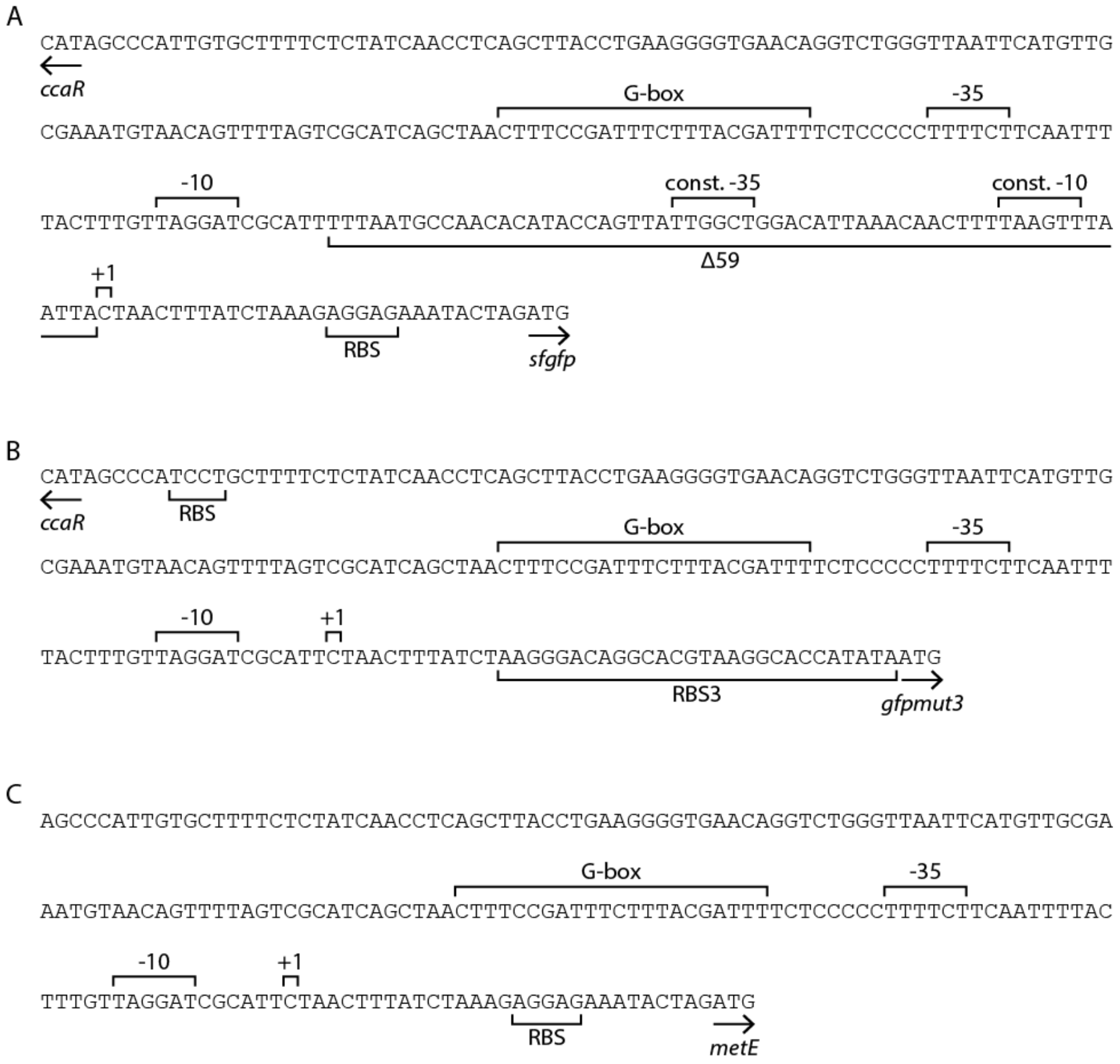

Supplementary Figure 1
Promoter sequences used in this study.

A. Original *cpcG2* promoter sequence from pJT119b [6], [11].
B. Plasmid pSKA413 *cpcG2Δ59* promoter sequence with synthetic *gfpmut3* RBS3 (RBS Library Calculator[12]) and *ccaR* 5'-AGGA-3' RBS.
C. Tn7 integration plasmid pSKA397 *cpcG2Δ59* promoter sequence.

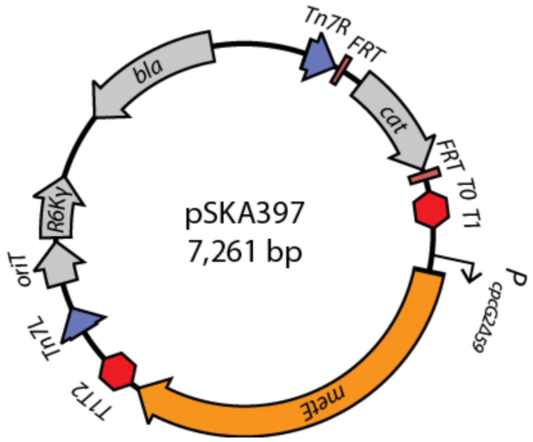

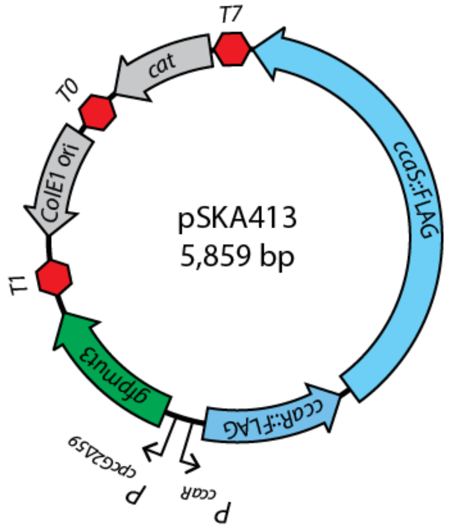

Supplementary Figure 2
Plasmid maps of pSKA413 and pSKA397.

Supplementary Figure 3

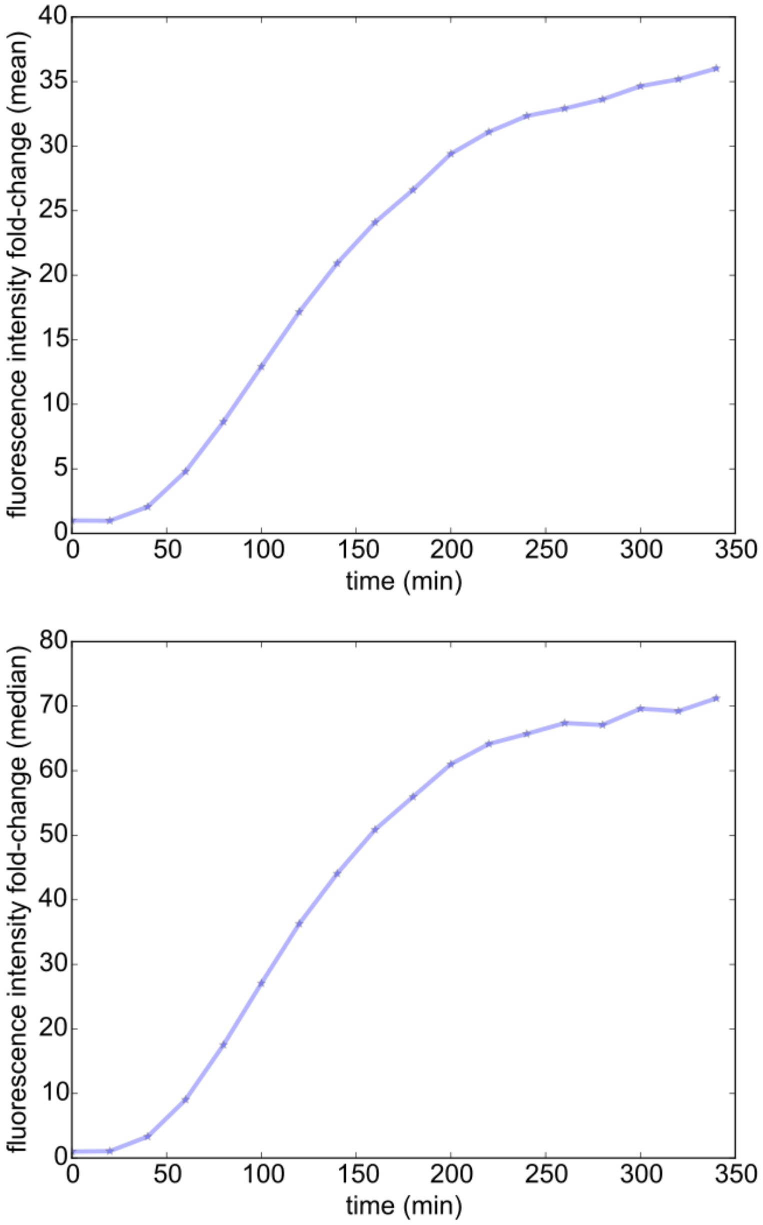

Step response of the JT2 strain with plasmids pPLPCB(S) and pSKA413. The strain was grown following the same protocol used for the GFP experiments (Methods of main text). It was subjected to maximal green light intensity and uniformly sampled by flow cytometry every 30 minutes, by means of the automatic setup described in the main text. The two plots correspond to the fold change in mean and median GFP abundance, respectively.

Supplementary Figure 4

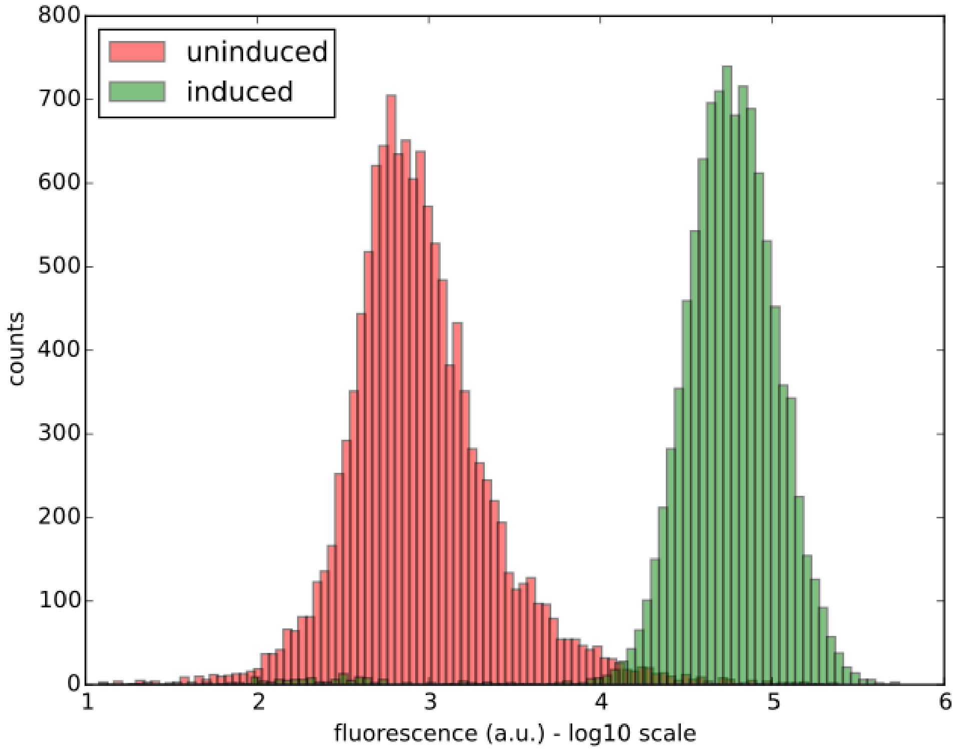

Flow cytometry distributions of the JT2 strain with plasmids pPLPCB(S) and pSKA413 corresponding to the first (red, uninduced) and last (green, induced) flow cytometry samples from Supplementary Figure 3.

Supplementary Table 1. Strains, plasmids, and primers used in this study.

**Strain**	**Description**	**Reference**
CC118 (λpir)	*Δ(ara-leu) araD ΔlacX74 galE galK phoA20 thi-1 rpsE rpoB argE(Am) recA1 λpir* phage lysogen	[15]
DL4259	MC4100 λ640-13	[10]
JT2	RU1012 ΔP_{ompC}-*lacZ*	[16]
MFDpir	MG1655 RP4-2 Tc::[ΔMu1::*aac*(3)IV-Δ*aph*A-Δ*nic*35-ΔMu2::*zeo*] Δ*dapA*::(*erm-pir*) Δ*recA*	[23]
NEB Turbo	F' *proA+B+ lacIq ΔlacZM15/fhuA2 Δ(lac-proAB) glnV galK16 galE15 R(zgb-210::*Tn*10)TetS endA1 thi-1 Δ(hsdS-mcrB)5*	New England Biolabs
SKA932	JT2 Δ*metE::cat*	This study
SKA936	JT2 Δ*metE::FRT*	This study
SKA973	JT2 Δ*metE::FRT Tn7::cat*-$P_{cpcG2\Delta59}$-*metE*	This study
SKA974	JT2 Δ*metE::FRT Tn7::FRT*-$P_{cpcG2\Delta59}$-*metE*	This study
**Plasmid**	**Description**	**Reference**
pCP20	FLP-recombinase plasmid, temperature sensitive, amp^R, cam^R	[19]
pJT119b	CcaR/CcaS green/red light-inducible plasmid, *ccaR, ccaS,* P_{cpcG2}-*sfgfp,* cam^R	[6]
pKD3	Plasmid with FRT-flanked chloramphenicol-resistance cassette, amp^R, cam^R	[14]
pKD13	Plasmid with FRT-flanked kanamycin-resistance cassette, amp^R, kan^R	[14]
pKOBEGA	Arabinose-inducible λ-Red recombinase plasmid, temperature sensitive, amp^R	[18]
pPLPCB(S)	PCB biosynthesis plasmid, $P_{lac/ara-1}$-*ho1-pcyA*, $spec^R$	[16]
pSKA373	pKD13*::cat*, amp^R, cam^R	This study
pSKA385	pUC18R6KT-mini-Tn7T-*cat(FRT)*, amp^R, cam^R	This study
pSKA396	pUC18R6KT-mini-Tn7T-*cat(FRT)*-P_{cpcG2}-*metE*, amp^R, cam^R	This study
pSKA397	pUC18R6KT-mini-Tn7T-*cat(FRT)*-$P_{cpcG2\Delta59}$-*metE*, amp^R, cam^R	This study
pSKA413	pZE3-$P_{cpcG2\Delta59\_RBS3}$-*gfpmut3*-P_{ccaR_RBS}-*ccaR::FLAG-ccaS::FLAG*, cam^R	This study
pTNS3	Tn7 transposase helper plasmid, amp^R	[22]
pUC18R6KT-mini-Tn7T	Mini Tn7 delivery vector, amp^R	[20]
**Primer**	**Sequence 5' > 3'**	**Reference**
84	CAACAAGCTAGCGCGGCCGCATTCCGGGGATCCGTCGACC	This study
85	CAACAAGCTAGCGTGTAGGCTGGAGCTGCTTC	This study
235	GGTTTTTCTACTGGGATTCGCTAATAT	This study
236	CTTGTCATCGTCGTCCTTGTAGTCAGCTCGAGGCAAATGGTTATAGCG	This study
240	TCTAGAGGCATCAAATAAAACGAAAG	This study
304	CTACTTGTCATCGTCGTCCTTGTAGTCGTTTTTCCCTTGGCACAAAG	This study
333	GGTACCGGGCCCAAGCTTCTCGAGAGAGTTTGTAGAAACGCAAAAAGG	This study
338	CTCTCCTGAGTAGGACAAATCC	This study
344	CTAGTATTTCTCCTCTTTAGATAAAGTTAG	This study
392	GGGGCCTCTAAACGGGTCTTGAGGGGTTTTTTGGACGTCGATATCTGGCGAAAATG	This study
393	CTTTCGTTTTATTTGATGCCTCTAGATTATTTGTATAGTTCATCCATGCCATG	This study
394	GCGAATCCCAGTAGAAAAACCCTACTTGTCATCGTCGTCCTTG	This study

395	CAAGACCCGTTTAGAGGCCCCAAGGGGTTATGCTAGTTACTTGTCATCGTCGTCCTTG	This study
449	CTAGTATTTCTCCTCTTTAGATAAAGTTAGAATGCGATCCTAACAAAGTAAAATTG	This study
451	TGGGCTATGAGAATTCTTTTAGTG	This study
459	CACTAAAAGAATTCTCATAGCCCATCCTGCTTTTCTCTATC	This study
461	CTAACTTTATCTAAAGAGGAGAAATACTAG	This study
514	GGATTTGTCCTACTCAGGAGAG	This study
536	CGATATCATGCATGAGCTCACTAGTAGCCCATTGTGCTTTTCTC	This study
551	CAACTTTTAAGTTTAATTACTAACTTTATCTAAAGAGGAGAAATACTAGATGACAATATTGAATCACACCCTCG	This study
558	CTAAATTCAAAATCCATAGGATTTACATATAATTAGAGGAAGAAAAAAATGATTCCGGGGATCCGTCGACC	[17]
559	CCGGGTGGTATTACCACCCGGTTTGGATTTTACCCCCGACGCAAGTTCTGTGTAGGCTGGAGCTGCTTCG	[17]
563	CAACAAGGATCCATGACAATATTGAATCACACCCTCG	This study
564	CAACAAGCATGCTTACCCCCGACGCAAGTTC	This study
565	CTTCGAACTGCAGGTCGAC	This study
566	ATTCCGGGGATCCGTCGACCTGCAGTTCGAAGTTCCTATTCTCTAGAAAGTATAGGAACTTCCAACTTTTGGCGAAAATGAGAC	This study
587	CAGCAGCACAGGTTTCAC	This study
597	TATATGGTGCCTTACGTGCCTGTCCCTTAGATAAAGTTAGAATGCGATCCTAAC	This study
598	ACAGGCACGTAAGGCACCATATAATGCGTAAAGGAGAAGAACTTTTC	This study

Supplementary Note 1. Turbidostat design

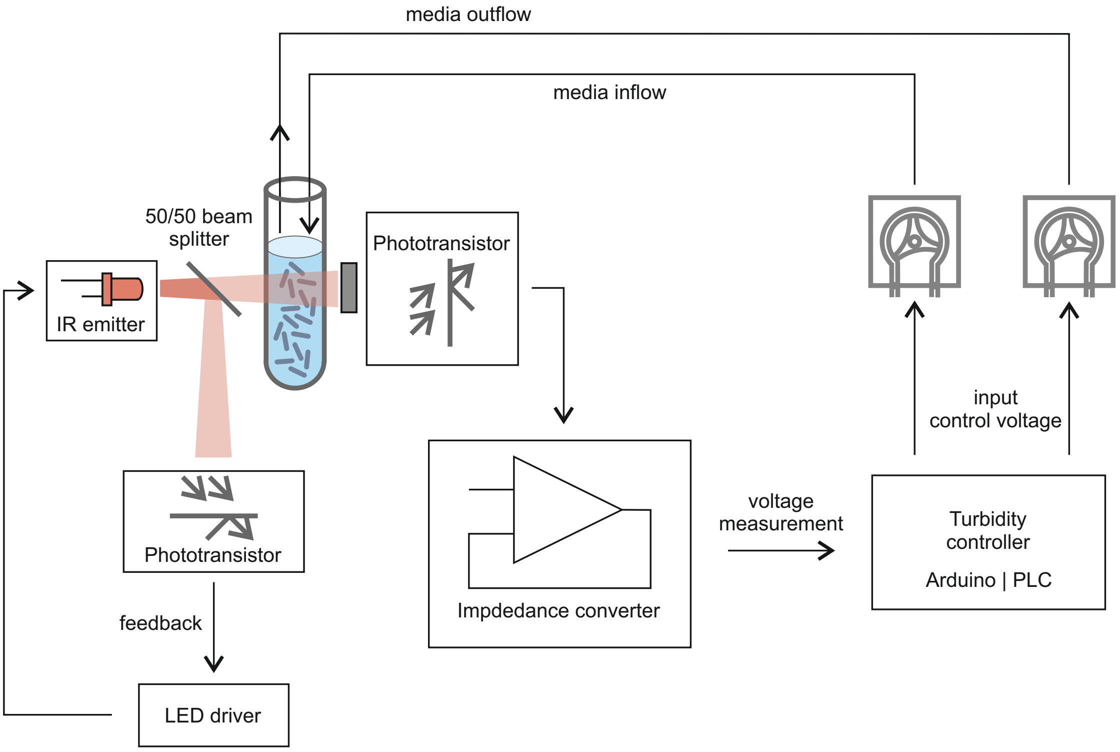

Figure S 1.1: Turbidostat schematic.

The turbidostat works by measuring the cell culture optical density and feeding it into a PI controller that computes the necessary medium influx rate to maintain a constant, user-defined culture density. The outflux rate is always set slightly higher than the influx rate, to ensure that the culture level remains at the height where the outflux tube is placed.

The optical density sensor contains an infrared LED that shines a beam through the culture, and a phototransistor that measures how much light reaches the other side of the glass tube. The light intensity output of the infrared LED is stabilized by the feedback of another phototransistor: 50% of the light emitted is deflected to the phototransistor by a beam divider mirror, while the other 50% of is transmitted through the cell culture to probe its turbidity. The light intensity reaching the phototransistor at the opposite end of the culture tube is converted to a voltage measurement via an impedance converter. This signal is read by a microcontroller and fed into the turbidity control algorithm, which dictates the pumps speeds.

Supplementary Note 2. Images of experimental setup

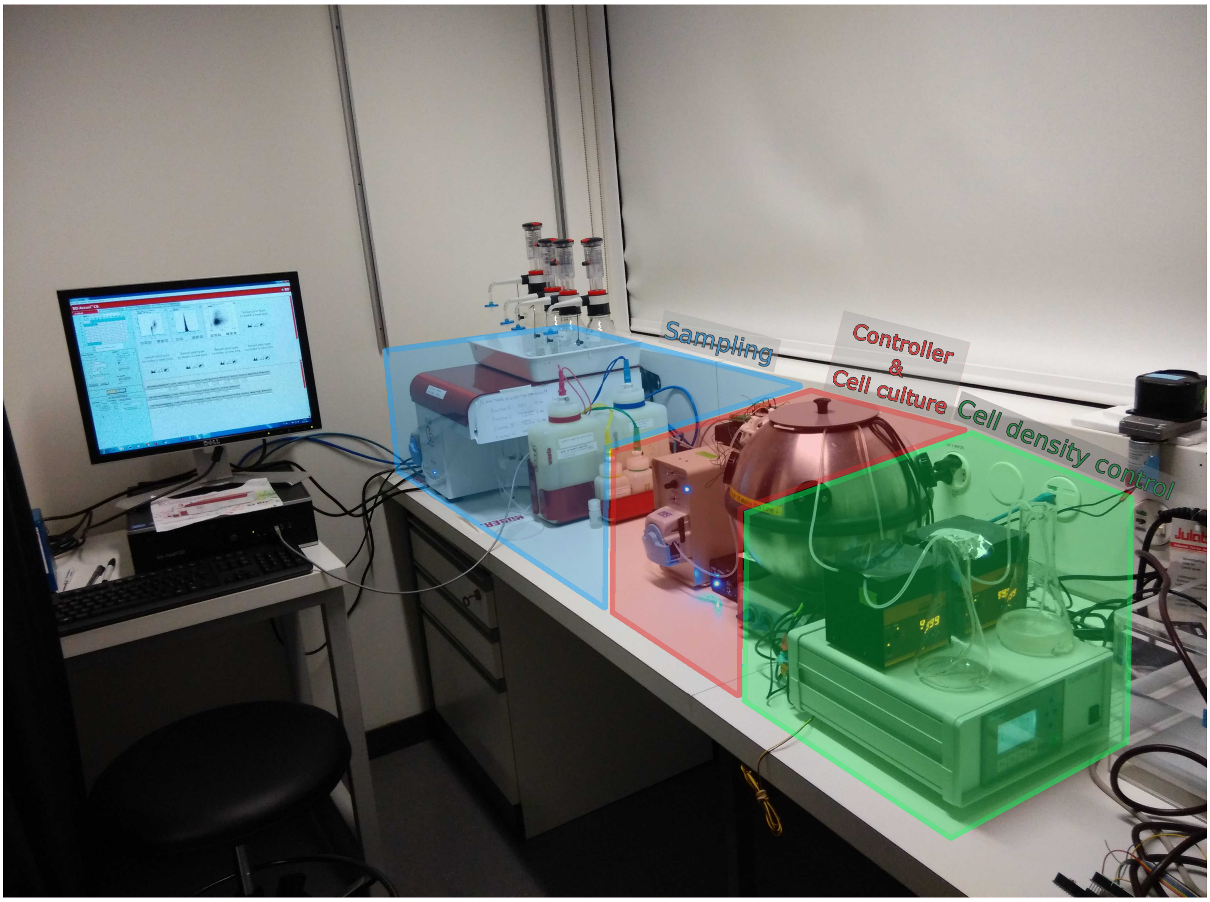

Figure S 2.1: Overview of the experimental setup.

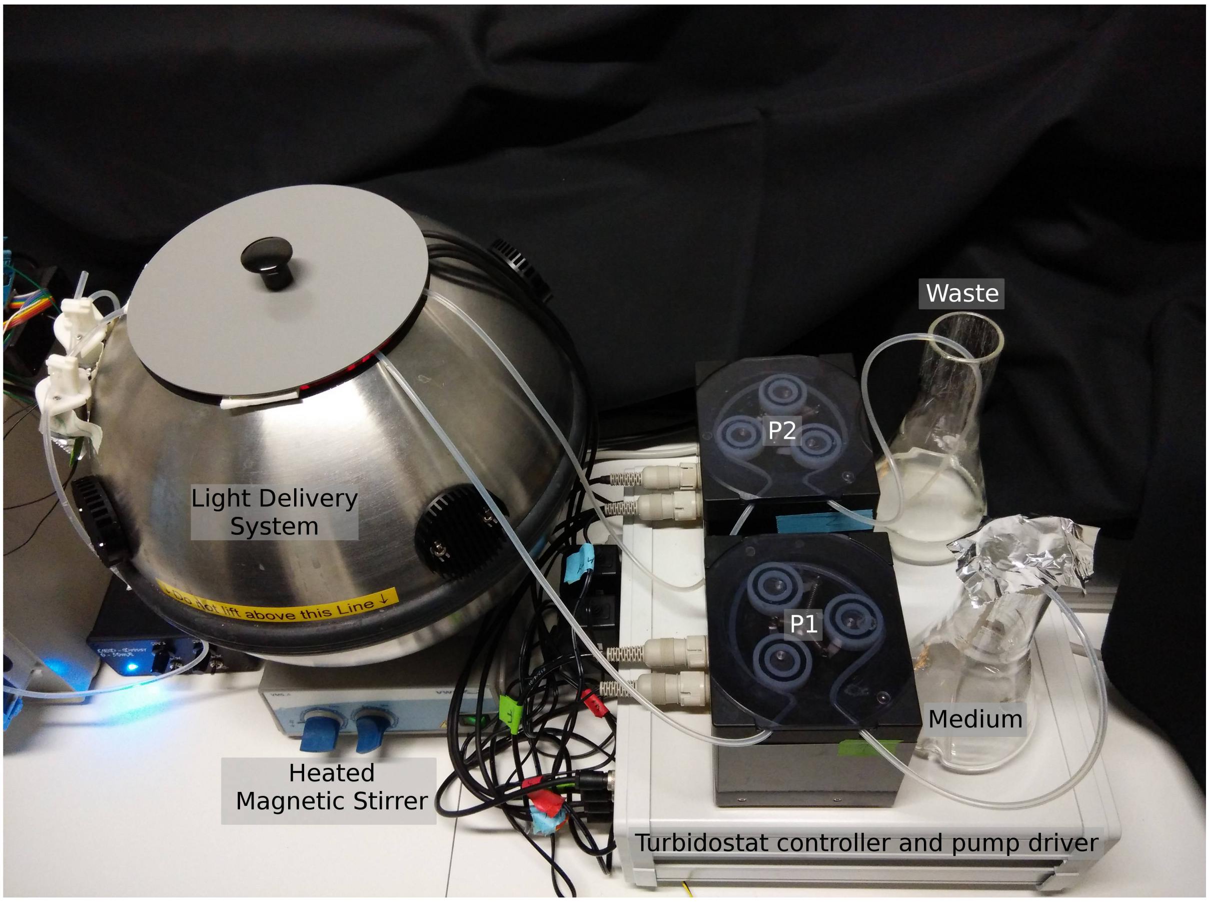

Figure S 2.2: Setup for cell density control.

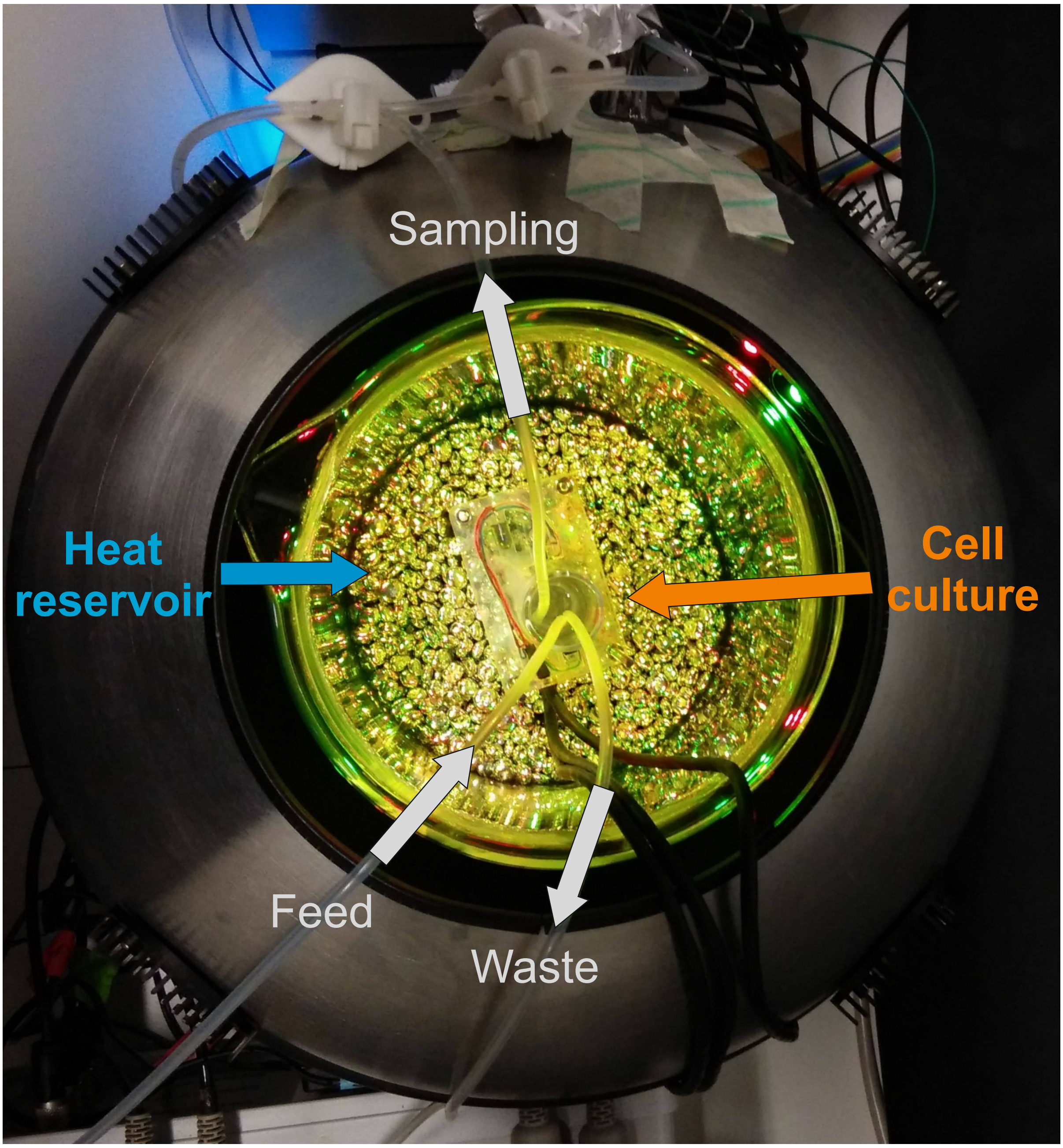

Figure S 2.3: Close-up image of the inside of the light delivery system, where the cell culture is grown under controlled temperature and stirring conditions and protected from external light. The culture is held inside a metallic beads bath that helps maintain a constant temperature.

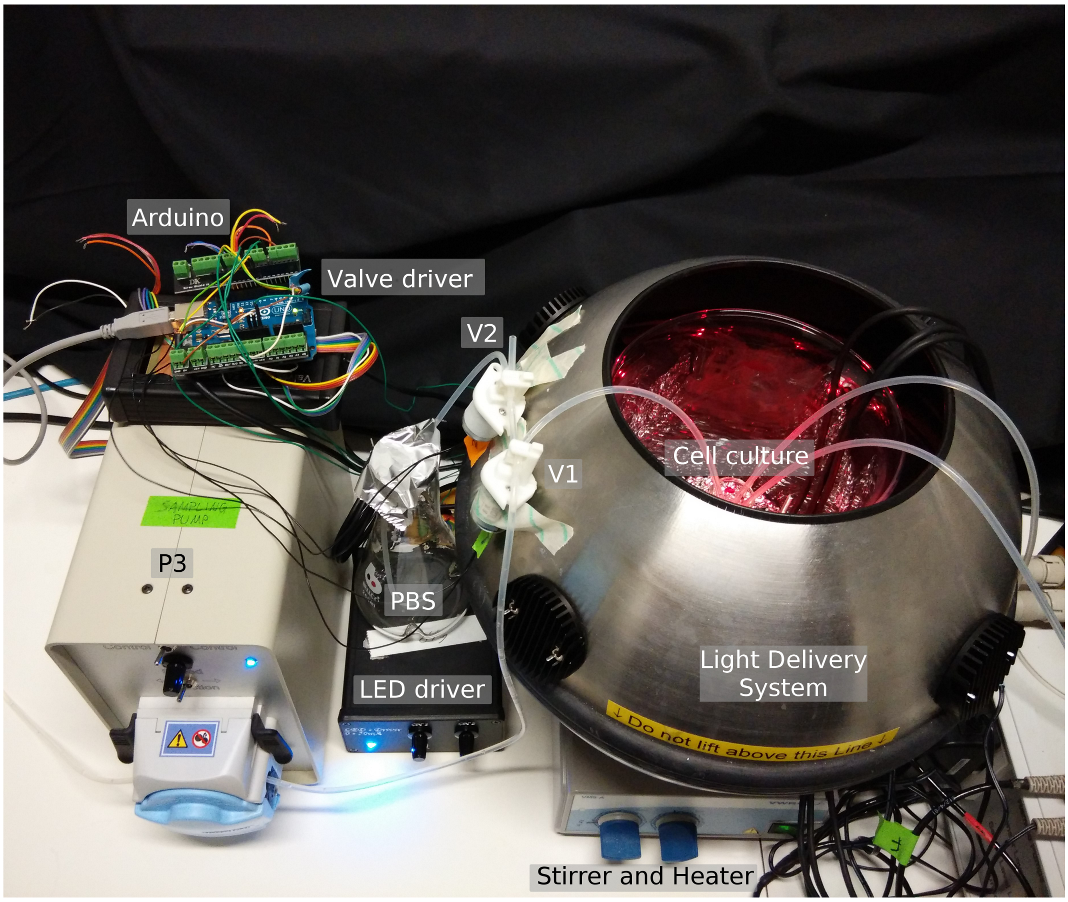

Figure S 2.4: Automatic setup for flow cytometry. Not displayed in this picture are the flow cytometer and the pump used to empty the flow cytometer sampling tube once the cells have been measured.

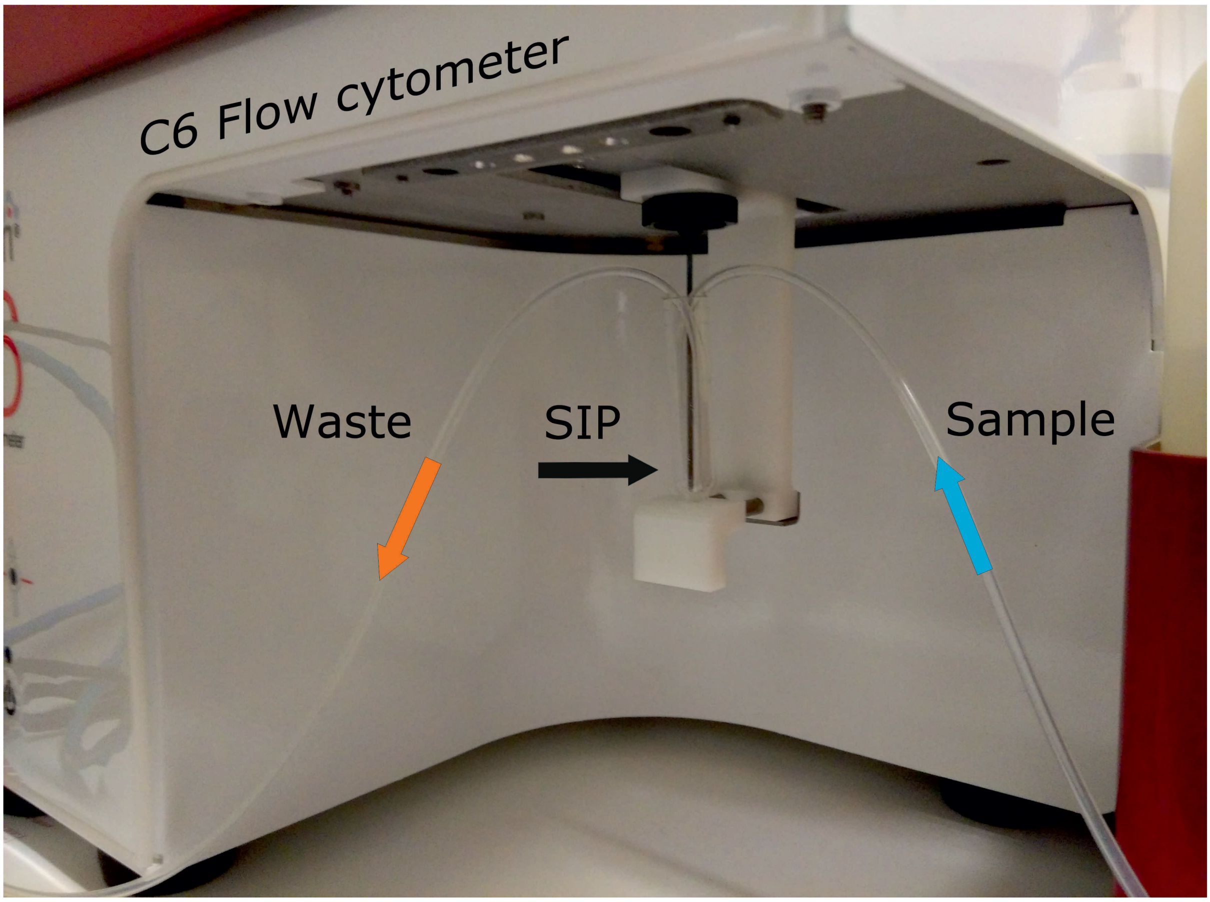

Figure S 2.5: A close-up of the flow cytometer sample introduction port (SIP).

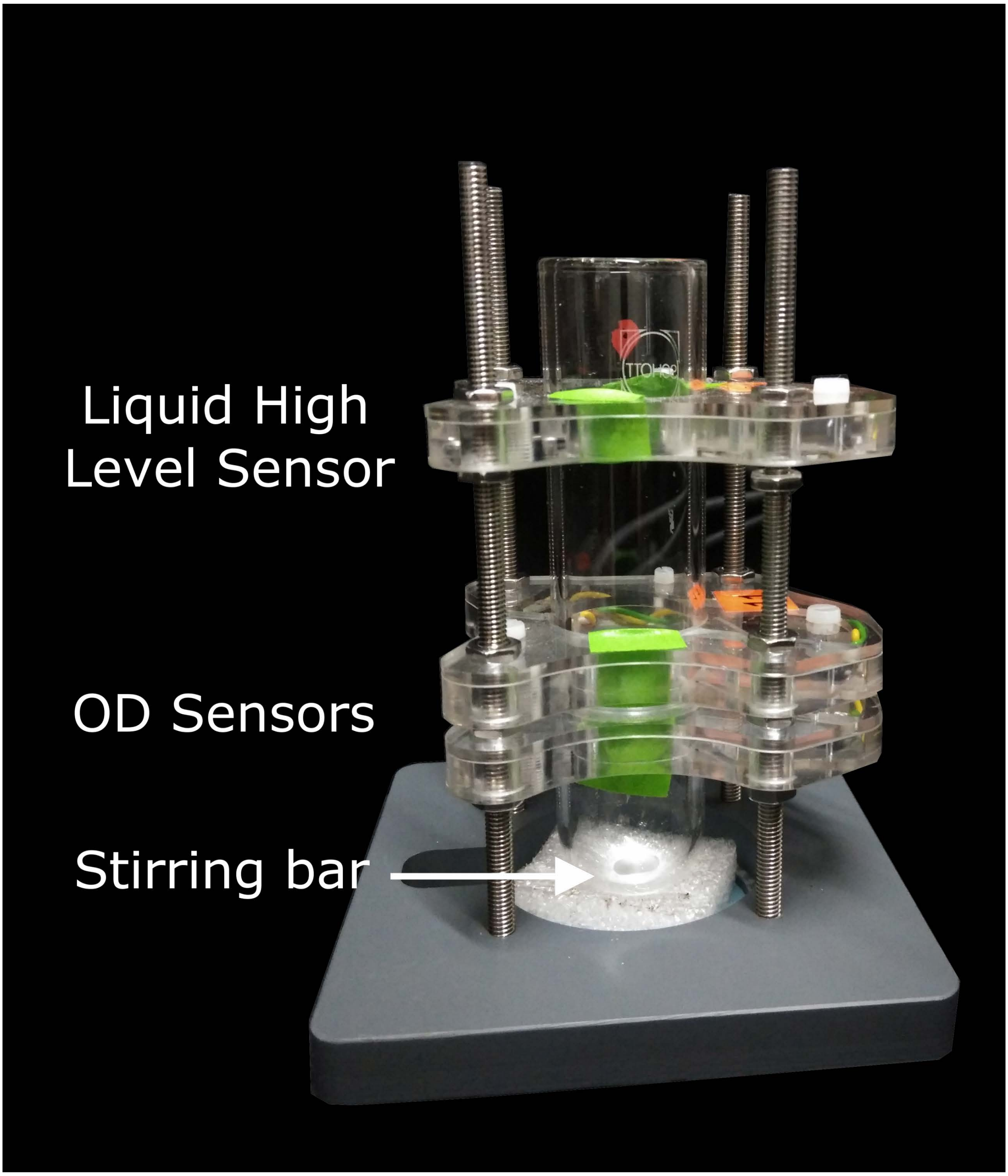

Figure S 2.6: Side-view of the cell culture glass.

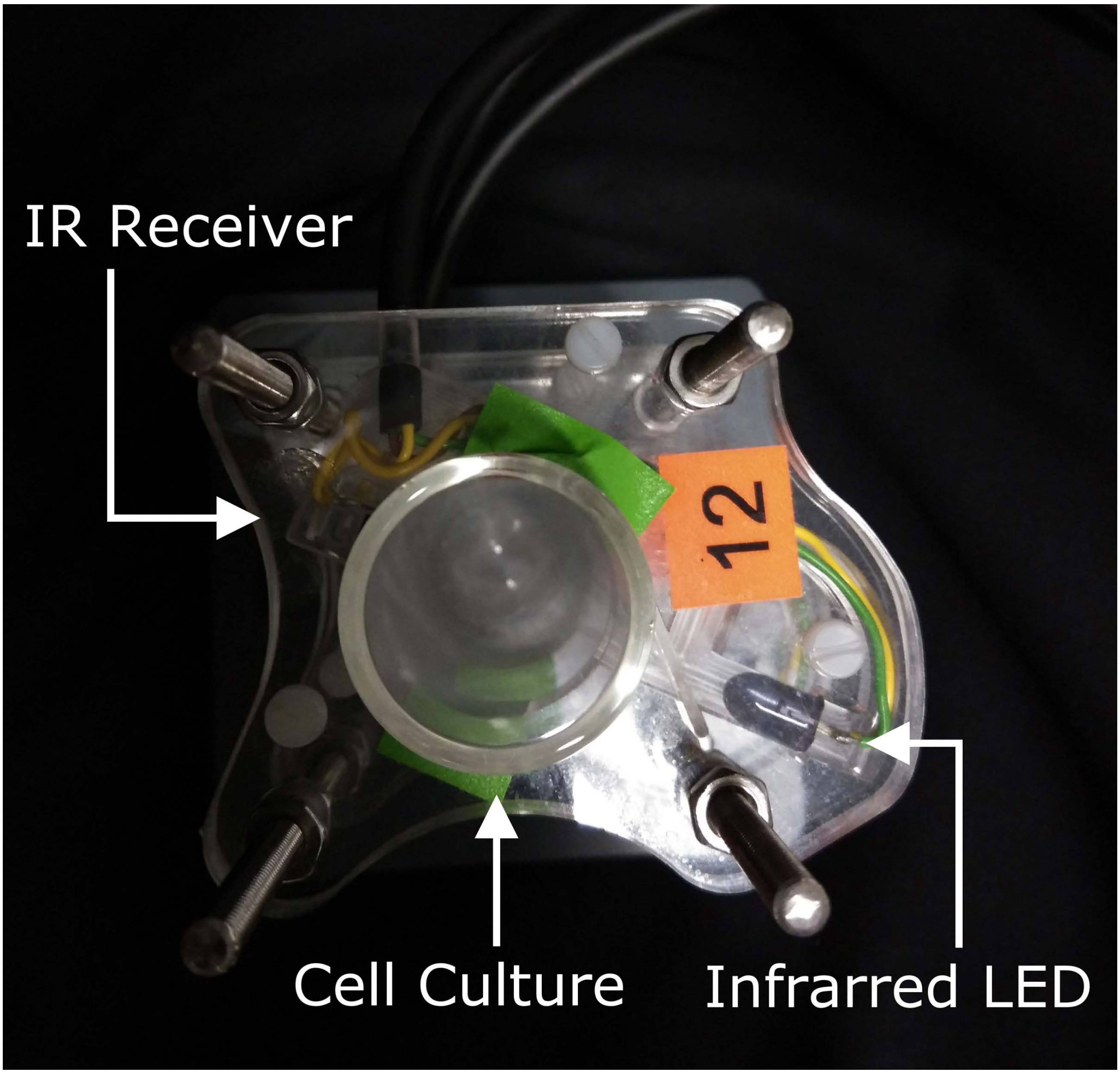

Figure S 2.7: Top-view of the cell culture glass showing the main components of the optical density sensor.

Supplementary Note 3. Day-to-day variability of background levels and dynamics of gene expression

It has already been observed that the CcaS-CcaR system displays non-trivial day-to-day variability in terms of background expression levels, despite the repeated application of the exact same growth protocol [6]. We have made similar observations, which are summarized in Figure S 3.1 below. The variability is observed both in normalized and unnormalized GFP means, and does not seem to particularly strongly correlated with FSC-H (which is used as proxy for cell volume).

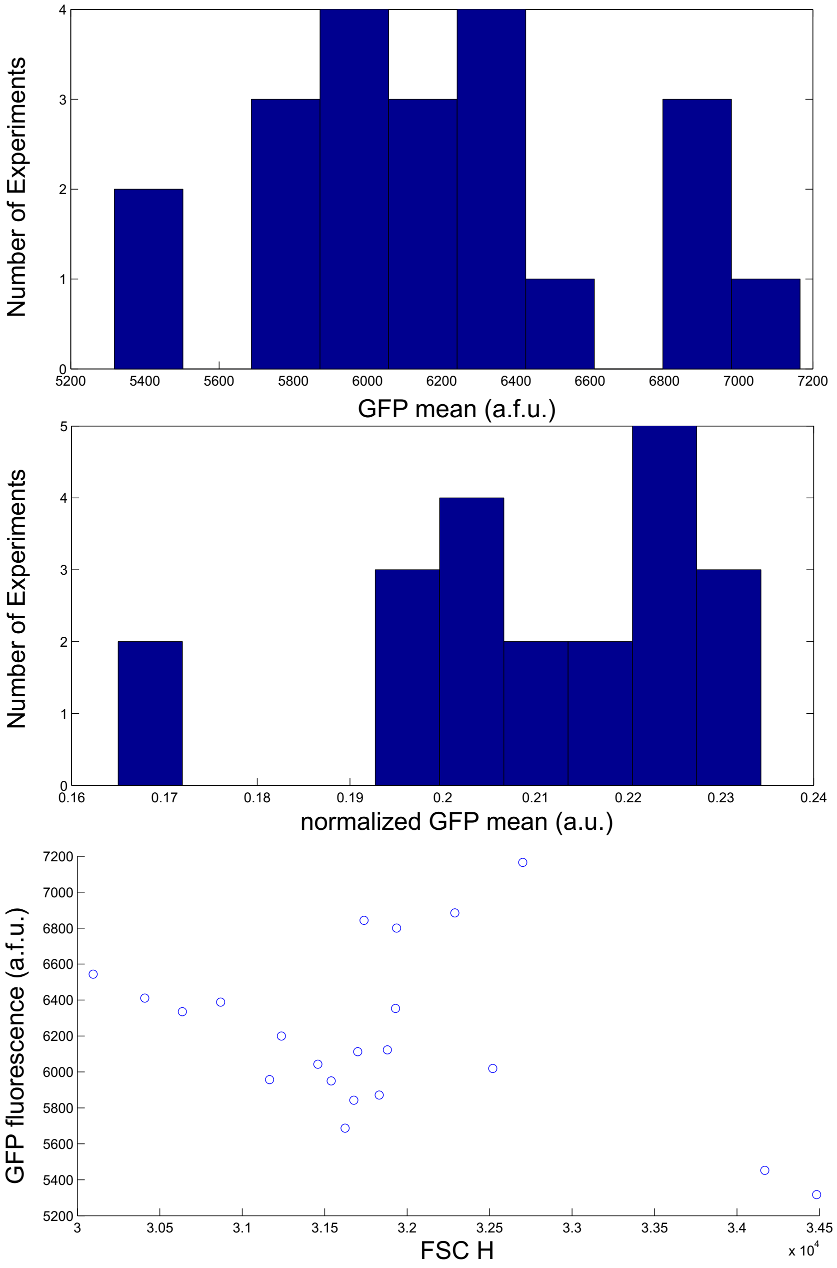

Figure S 3.1: Summary of initial GFP fluorescence levels of dark-grown overnight cultures. The histograms compile data from 21 different cultures.

The maximum GFP fold change has been also found to be variable from day to day [6]. We have additionally observed that even the speed at which the system responds to a step change in the green light intensity (from zero to full power) is variable from day to day. Consequently, not only the steady-state gain of the system, but also its dynamics are also variable. This behavior is shown on Figure S 3.2.

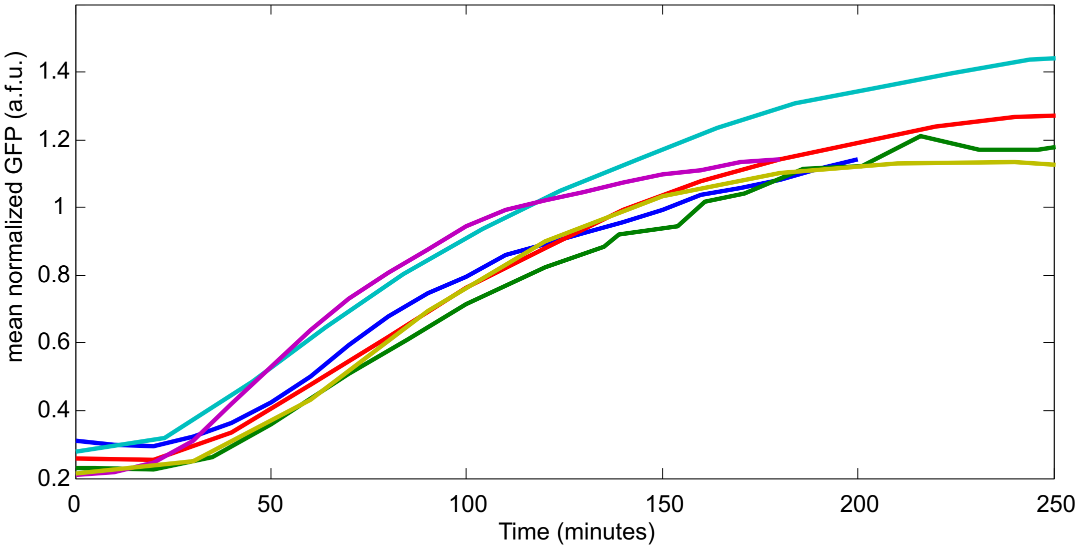

Figure S 3.2: System response to a step change in green light input from zero to full. Different curves correspond to responses measured on different days, from different overnight cultures.

Supplementary Note 4. PI controller tuning

Overview of the approach: Although several automatic tuning methods for PI controllers exist in the automatic control literature [1], they are only applicable to linear systems. Since the CcaS-CcaR system is manifestly nonlinear, PI tuning using one of these methods would not be optimal. The PI controllers used in this work were therefore tuned by a trial-and-error method: first, using the fitted linear model, we got rough estimates of the gains through simulation. Afterwards, using these gains as starting points, we fine-tuned them experimentally by observing the tracking performance of the controller for a certain constant reference, in order to maximize convergence speed to the target and minimize overshoot and oscillations. After a couple of iterations, the gains $K_P = 80$ and $K_I = 8$ were found to yield good results for tracking a constant reference, and they were used to generate the plots of Figure 2 in the paper. For Figure 3, the same gains were used during the first tracking phase, and were increased to $K_P = 160$ and $K_I = 20$ after the given reference was achieved, to maximize the disturbance rejection performance of the controller.

Linear model results: To get an initial idea of the required PI controller gains, we used the simple fold-change linear model with input delay described previously, with the additional limiting of input values within $[0, 4]$ (cf. with discussion of the MPC setup). The tracking of a constant reference by PI controller is affected by the ratio and absolute magnitudes of K_P to K_I. If the gains are chosen too large, the system output will oscillate wildly; if they are chosen too small, convergence will be very slow. Figure S 4.1 shows two extreme situations, where the gains are chosen with a constant ratio but different absolute magnitudes.

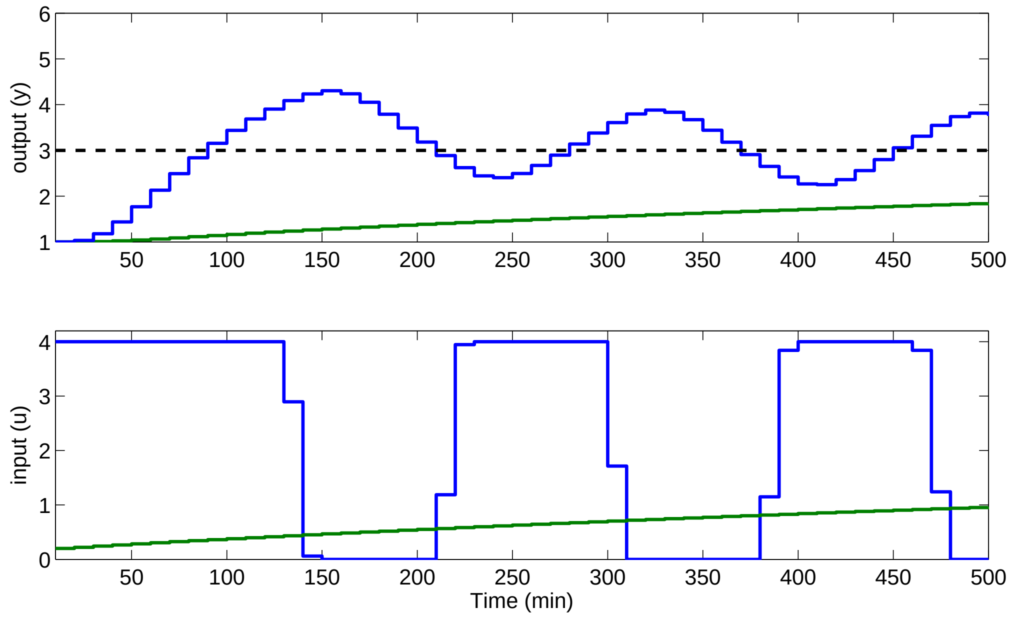

Figure S 4.1: Simulation of the fold-change system in a feedback loop with a PI controller, using two sets of improper PI gains. Blue line: $K_P = 1000$, $K_I = 110$. Green line: $K_P = 10, K_I = 1.1$. Observe that input values are saturated from below at 0 and from above at 4. The dashed black line denotes the desired constant output level.

On the other hand, for a fixed value of K_P, variation of K_I results in the responses shown on Figure S 4.2. In this case, the intermediate values $K_P = 100$, $K_I = 11$ seem to result in fast convergence with very little overshoot. As the results of Figure S 4.2 suggest, a K_P/K_I ratio of about 10 is optimal for our system.

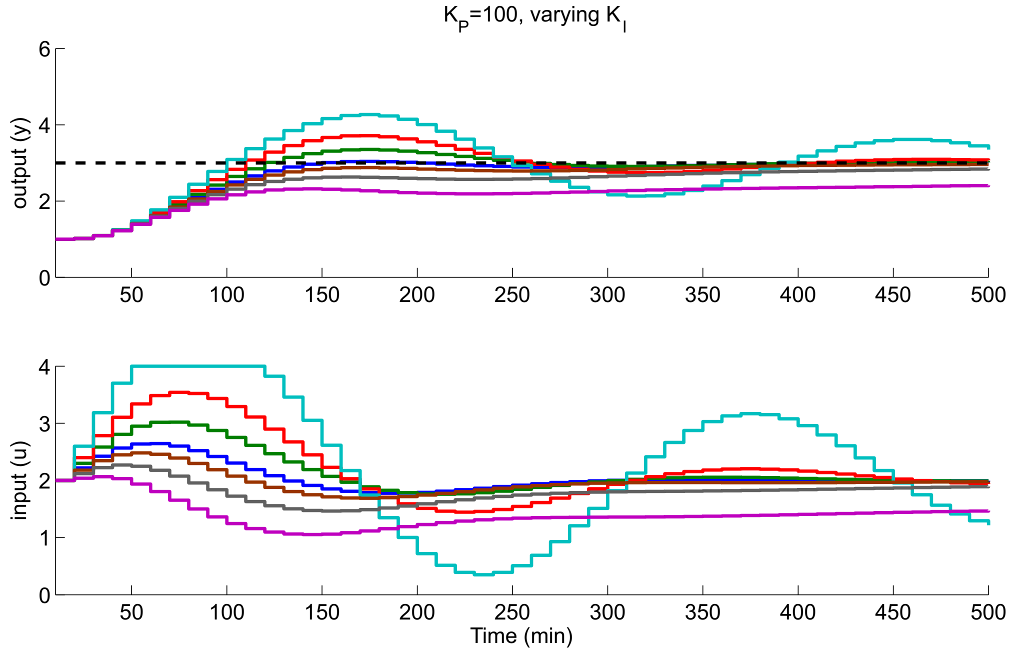

Figure S 4.2: Closed-loop simulation of the fold-change system with a PI controller. K_P is fixed and K_I is varied. Blue: $K_I = 11$. Green: $K_I = 15$, Red: $K_I = 20$. Cyan: $K_I = 30$. Brown: $K_I = 8$. Grey: $K_I = 6$. Magenta: $K_I = 2$. As K_I grows, overshoot (and eventually) damped oscillations begin to appear. On the other hand, small values of K_I result in undershoot and very slow convergence to the setpoint.

Supplementary Note 5. Additional input profiles for Fig. 2

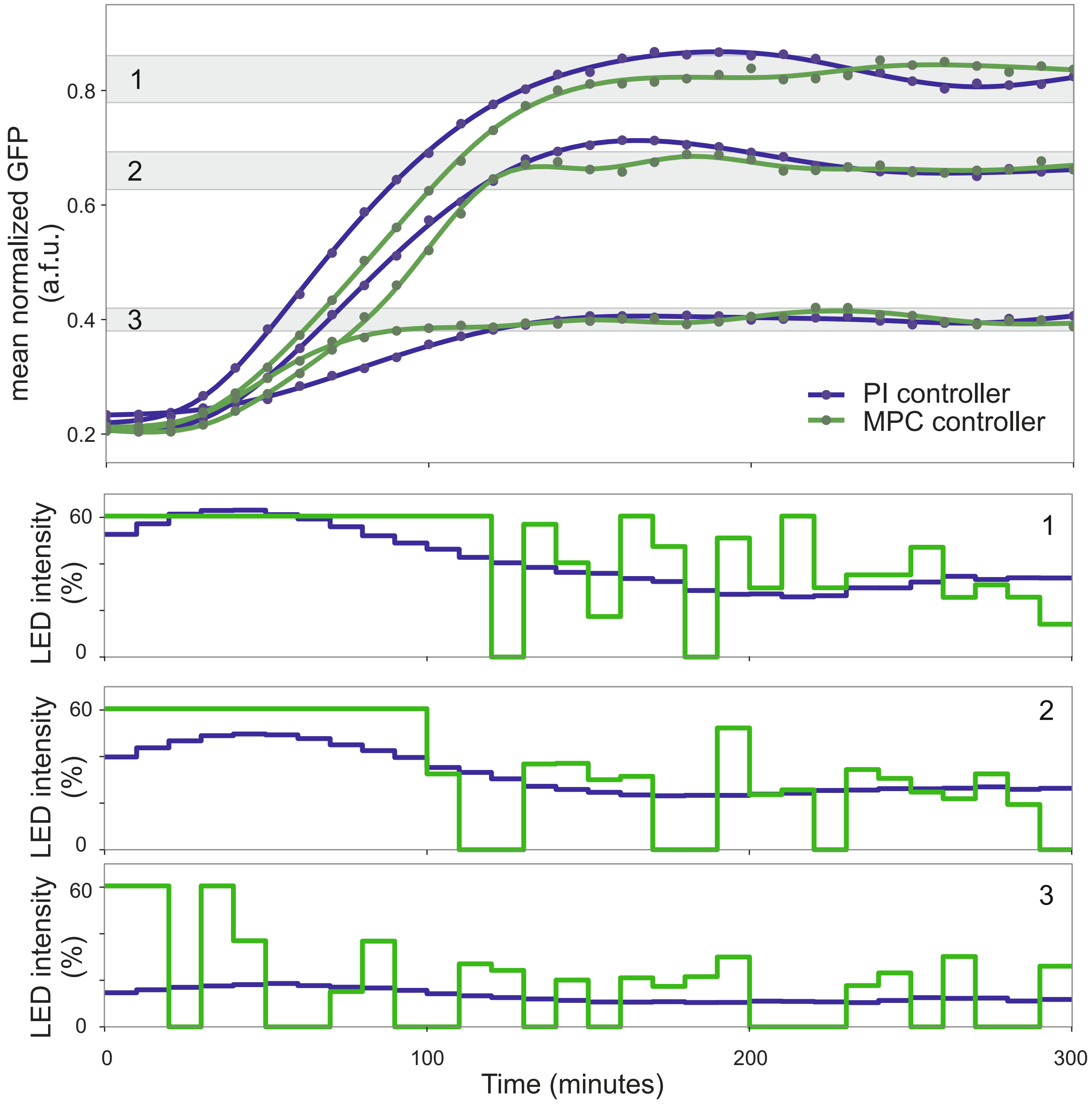

Figure S 5.1: **Constant reference tracking**: PI and MPC controller responses (purple and green lines, respectively) set to track different references (0.4, 0.66, 0.82 a.f.u.). Dots represent experimental samples and the lines are polynomial fits. The grey shaded areas encompass +/- 5% of the reference values. The numbers on the left side of each grey-shaded box indicate the input used to generate the responses, represented below. The plot contains the additional input profiles that are not reported on Figure 2.

Supplementary Note 6. Cell size response to environmental changes

Changes in FSC-H (proxy for cell volume) due to environmental changes. In the first plot, media is progressively switched from M9 to LB at the 300 minute mark. The second plot shows the change in cell size due to a temperature change from 37°C to 28°C at the 220 minute mark. The different lines correspond to experimental replicates performed on different days.

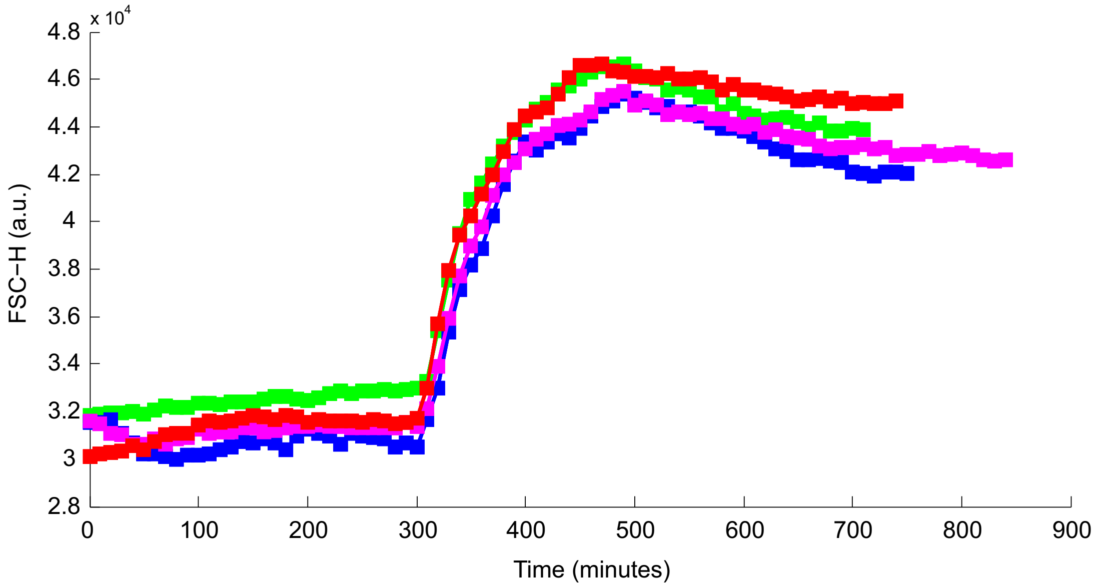

Figure S 6.1: Changes in cell size after a shift from M9 to LB.

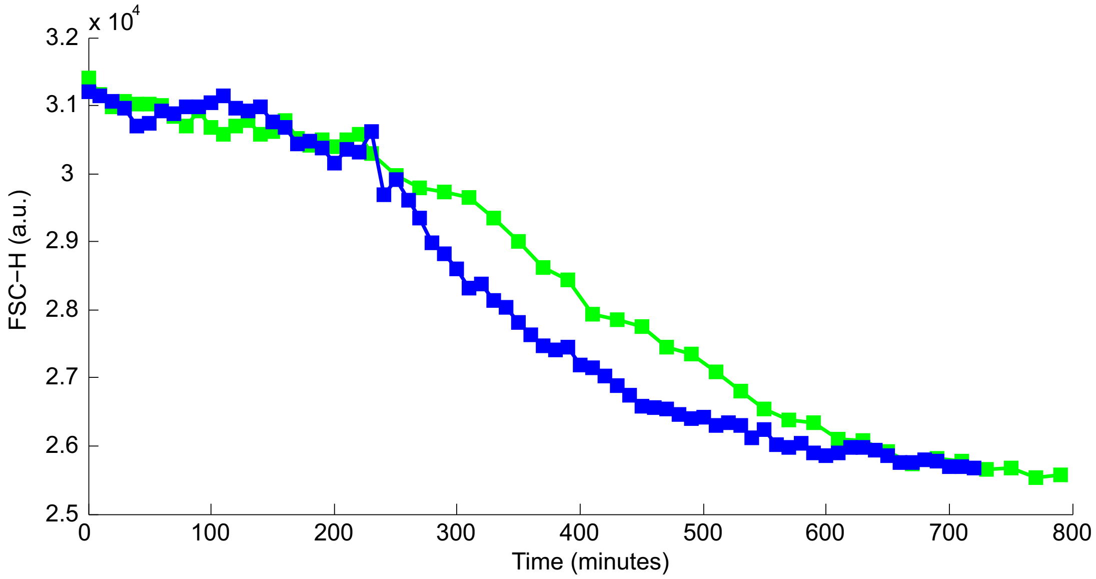

Figure S 6.2: Changes in cell size after a shift from 37°C to 28°C

Supplementary Note 7. Growth rate control

7.1 Data processing

Our strategy for estimating the culture growth rate makes use of the influx rate of fresh media required to maintain a constant optical density (OD) of the culture. As described in the main paper, the culture OD is fed into the turbidostat PI controller, which acts back on the cell culture by manipulating the flow rate of the pumps. The equations governing the change in optical density of the culture are:

$$\frac{d(OD)}{dt} = OD(\mu - \frac{u}{v}) \implies$$
$$\mu = \frac{u}{v} + \frac{1}{OD}\frac{d(OD)}{dt} \approx \frac{u}{v}$$

where μ is the growth rate, v is the culture volume [ml], and u is the influx rate $[\frac{ml}{min}]$. The influx rate in turn, is computed by converting the control voltage that is applied to the pump into flow rate using a regression line obtained from calibration tests (the pumps display a perfectly linear relationship between control voltage and flow rate). Finally, when the OD is tightly regulated, its time derivative is negligible and the culture growth rate is proportional to the medium influx rate, u.

However, the signal from the turbidity sensor is quite noisy, which implies that the signal coming out of the turbidity controller is also contaminated with noise and needs to be filtered before being fed into the growth rate controller. For this reason, we employ a smoothing algorithm based on local linear regression. The smoother uses the 500 most recent samples of the pump speed (gathered within 10 minutes, at sampling frequency of 0.83 Hz) to derive a local linear fit and take the midpoint of the resulting line as an estimate of the pump speed at the current time. This operation introduces a delay of 5 minutes in the pump speed estimate, but its effect is negligible, since the controlled system (cell growth rate) has much slower dynamics. The filtering is further improved post-experiment for plotting purposes by taking a moving average of the unfiltered pump speed signal with a sliding window of 2500 samples. This is the signal plotted on Fig 4 of the main text.

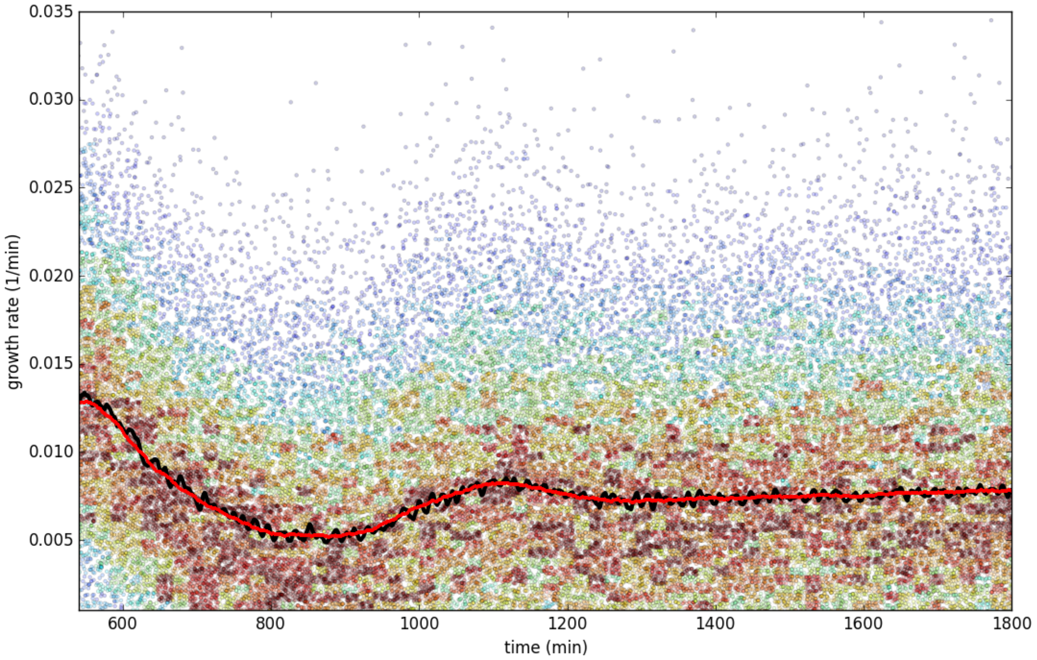

Figure S 7.1: An example of the raw and processed growth rate data: dots represent instantaneous estimates obtained by dividing the measured pump speed (u) with the culture volume (v). Dots are colored according to the histogram frequencies of the binned signal. The black line shows the signal obtained from local linear regression, which is fed into the growth rate controller, while the red line shows the smoothed signal from the moving average filter, obtained after the experiment.

7.2 Dynamic range

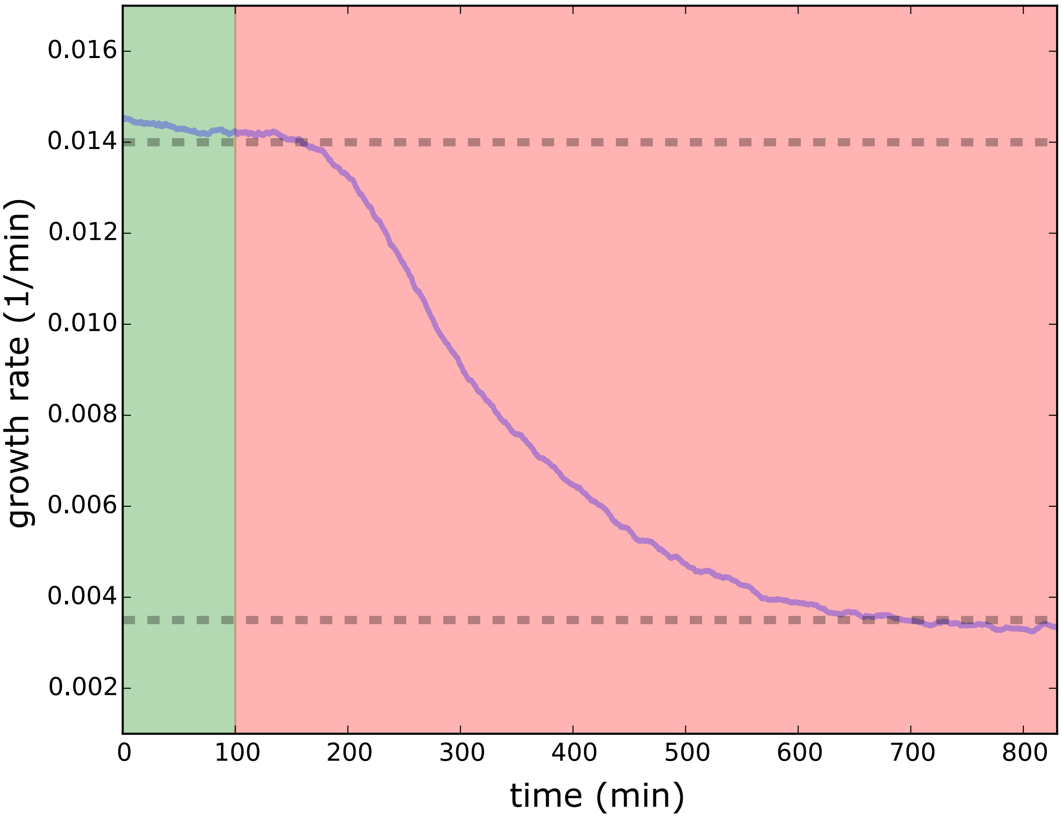

Figure S 7.2: Red light step response. Cells initialized under green light overnight and growing at maximal speed are switched to constant red light (at t = 100 min). The growth rate falls from about 0.014 min^{-1} to 0.0035 min^{-1}. This corresponds to a transition from a doubling time of around 50 min to a doubling time of 200 min. The dotted grey lines indicate the minimal and maximal growth rates that this strain displays.

7.3 Growth rate drift

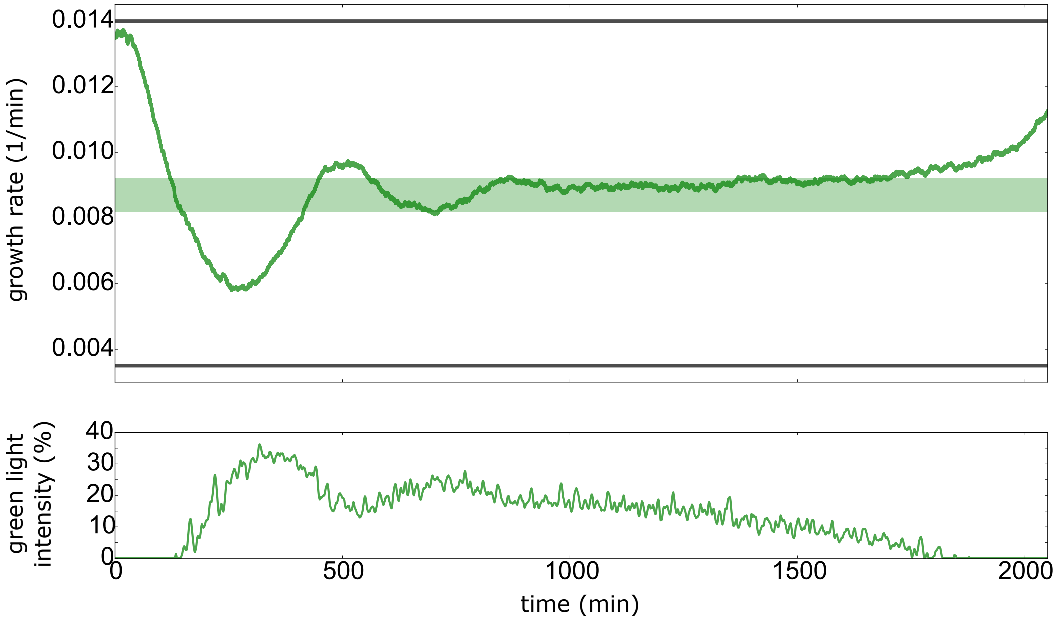

Figure S 7.3: Closed-loop setpoint tracking experiment aimed at maintaining the growth rate at 0.0087 min^{-1}. Starting at around 1250 min, the cells display a gradual loss of sensitivity to the light inputs received (they require less light to grow at the same rate). This can be seen by the negative slope of the green light intensity. Even though the cell culture is gradually becoming insensitive to light, its growth rate changes minimally because of the controller action, which opposes this change. However, at 1800 min the actuation boundaries of the controller are met: the green light intensity hits 0%, while the red light intensity is maximal. Beyond that point the controller is unable to act on the cells, and the culture shows a rapid increase in growth rate.

7.4 Additional experiments

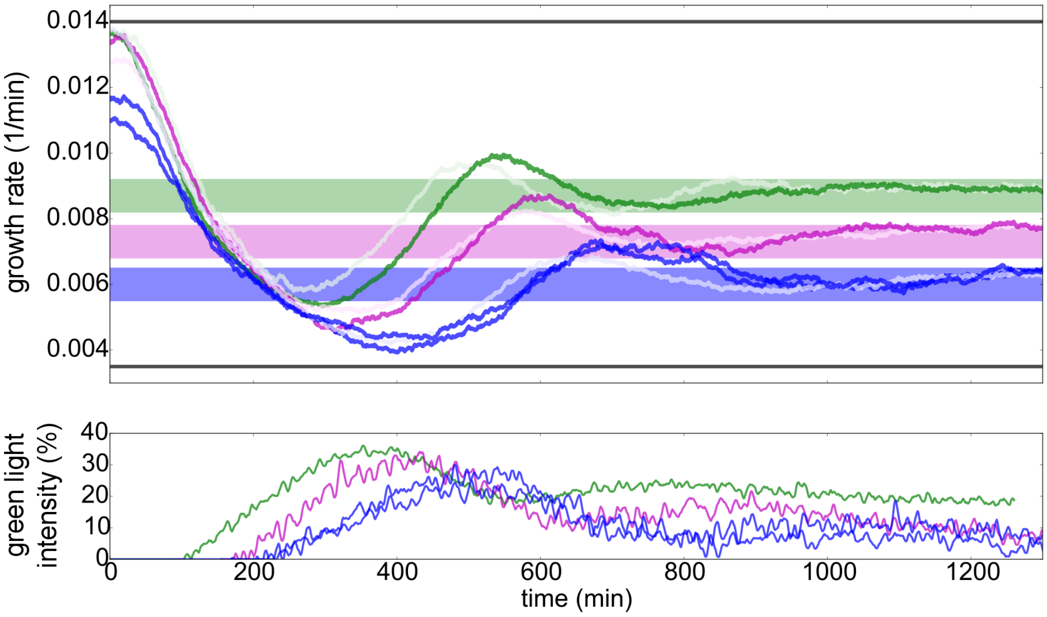

Figure S 7.4: Summary of additional closed-loop growth control experiments. The light grey lines correspond to the experiments presented on Fig. 4 of the main text. The green, magenta and blue curves track constant growth rates of 0.0087, 0.0073 and 0.006 min^{-1}, respectively. The colored lines all used the same PI controller, with gains $K_I = 45, K_P = 5700$.

Supplementary Note 8. Accuracy of the autosampling system

8.1 Cross-sample contamination

According to the automatic sampling protocol described in Online Methods, the silicone tubing of our system, as well as the sampling tube, are rinsed with PBS before the measurement of each sample in the cytometer. To determine the cross-sample contamination of the system, i.e. what percentage of a previously run sample is contained within the currently acquired, we ran a sequence of alternating culture and filtered water measurements.

In this test, a culture sample was first acquired. The piece of silicone tubing immersed in the culture was subsequently taken out, cleaned externally with ethanol, and put inside a tube with filtered water, to acquire a water sample. We then compared the cell counts per second that pass our gating criteria (see Online Methods) in the culture and water samples. Table 8.1 below summarizes the results obtained from three repetitions of this procedure.

	Replicate number			
	1	2	3	Mean
Culture sample counts	1150	991.5	1037	1060
Water sample counts	11	27	20	19

Table S 8.0: Cross-contamination in the automatic sampling system, measured by consecutive runs of culture and water samples.

Taking into account the fact that a water sample normally results in zero events inside our gates, the cross-sample contamination of our automatic system is about 2%.

8.2 Comparison with manual sampling

To verify that our setup's measurements are comparable with the ones obtained by manual sampling we performed a series of measurements where a cell culture was sampled both automatically and manually (10 μL of culture diluted in 100 μL of PBS). The cytometer was backflushed between each measurement. Table 8.2 below summarizes the results from seven repetitions of this procedure.

	Replicate number								
	1	2	3	4	5	6	7	Mean	s.d.
Manual (norm. GFP)	0.1814	0.1778	0.1775	0.1769	0.1736	0.1808	0.1786	0.1781	0.0026
Automatic (norm. GFP)	0.1826	0.1816	0.1771	0.1793	0.1784	0.1785	0.1786	0.1794	0.002

Table S 8.0: Comparison of manual vs. automatic sampling for the normalized GFP mean. According to a two-sample t-test, the null hypothesis (the means of manual and automatic samples are the same) is not rejected at a significance level of 5%.

Supplementary Note 9. Flow cytometry data processing

9.1 Gating procedure

As described in the Methods section, the raw flow cytometry data is processed by first selecting all events with a forward scatter value greater than 11.000 (a.u.), and then gating the remaining events with an elliptical gate.

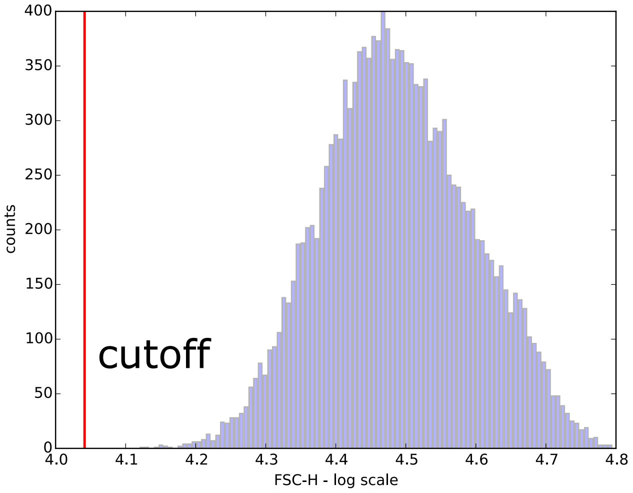

Figure S 9.1: Forward scatter distribution of E. Coli cells. The red line indicates the particle size cutoff value of 11.000 (a.u.).

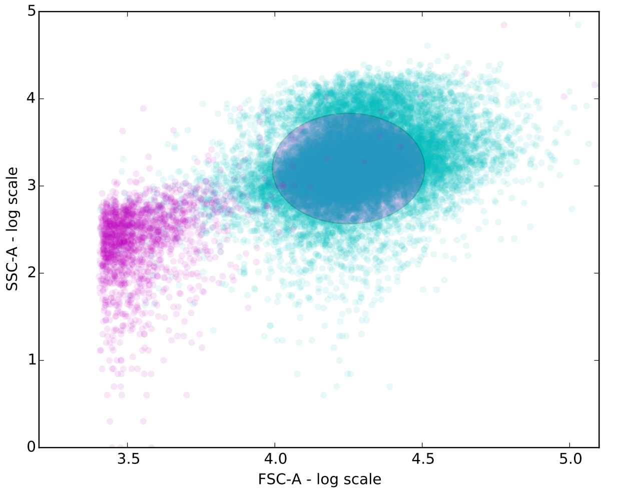

Figure S 9.2: Depiction of the elliptical gate in the FSC-A vs. SSC-A space used to process the flow cytometry data in conjunction with the fluorescence threshold. Magenta points correspond to events with a lower FL1 (GFP fluorescence channel) value than the established threshold (800 a.u.). This threshold does not significantly affect the cells that end up in our elliptical gate. It is done to remove part of the background flow cytometry noise.

9.2 Evolution of flow cytometry histograms over time

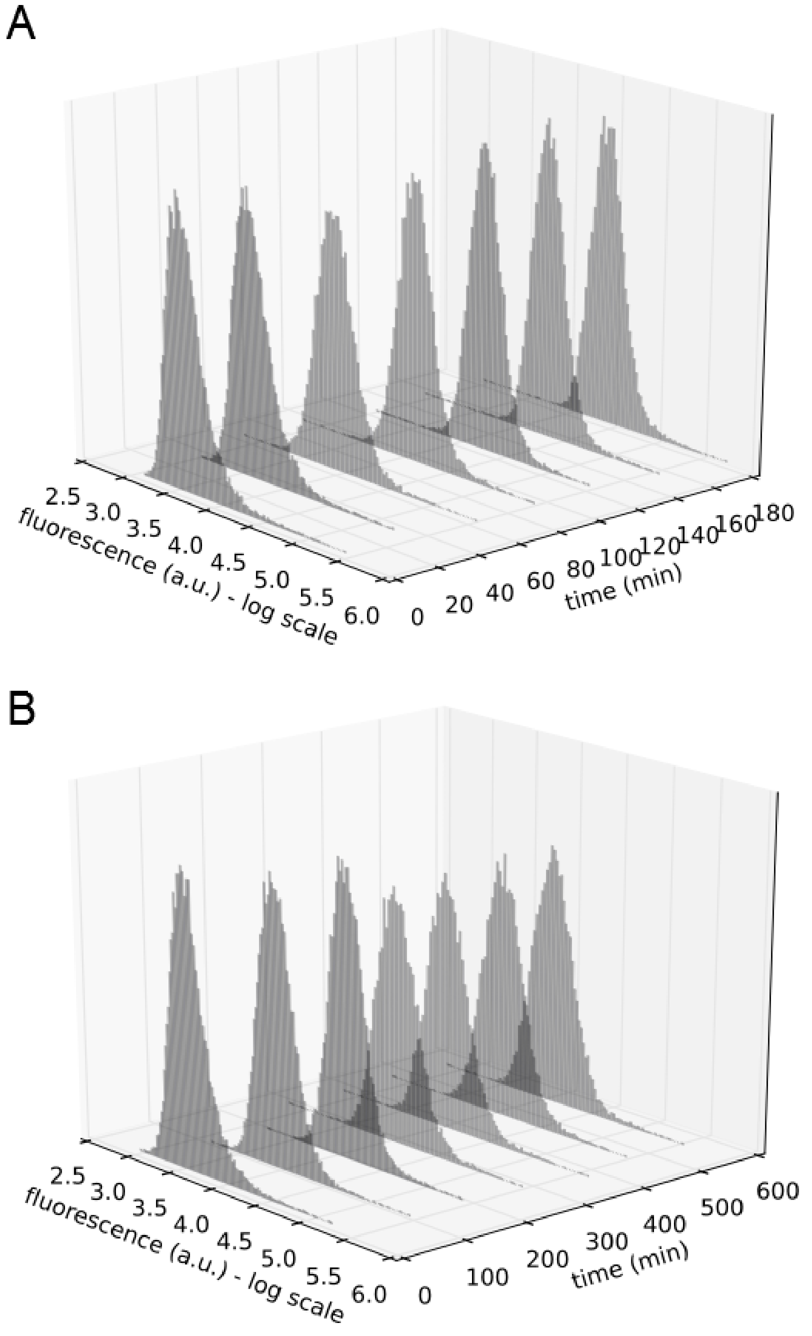

Figure S 9.3: Example flow cytometry distributions over time. A. Step response experiment (open loop) B. Closed loop experiment (MPC controller).

Supplementary Note 10. Need for gain scheduling

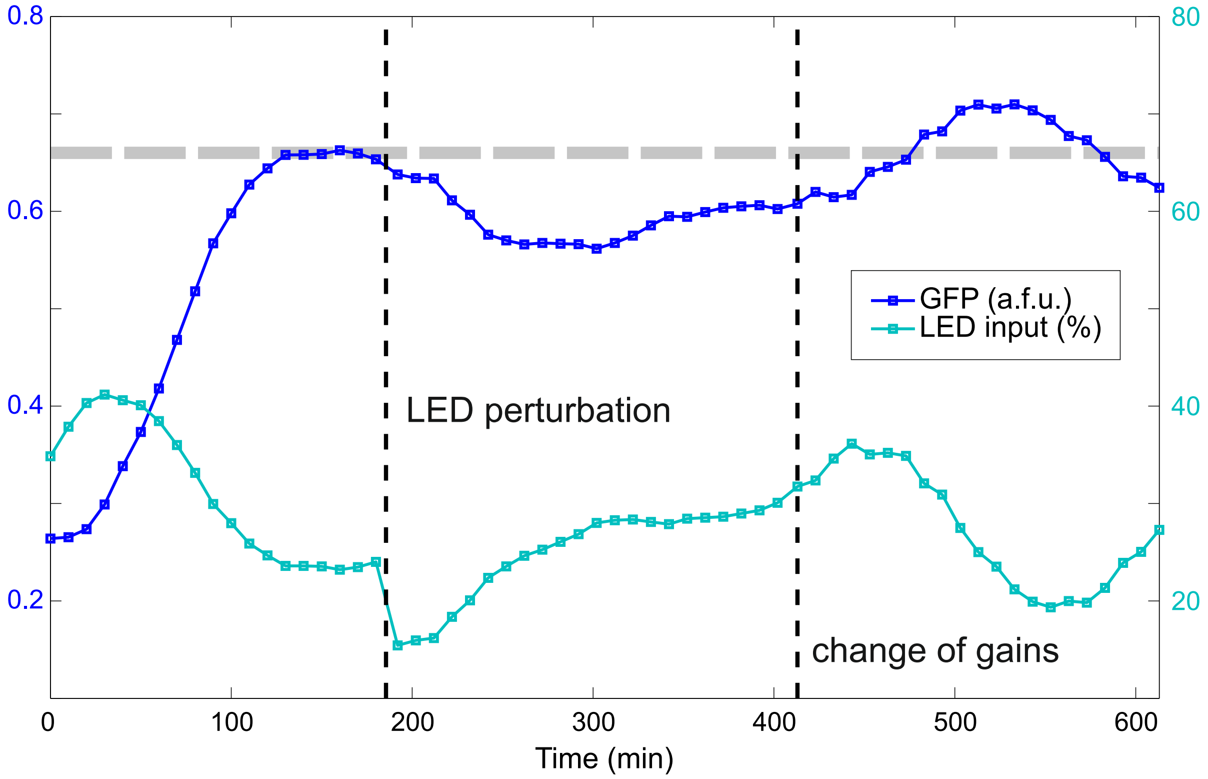

Figure S 10.1: Rejection of an input perturbation using a PI controller. The first phase of the experiment corresponds to a PI controller tracking a constant setpoint (grey dashed line). In the second phase of the experiment (after 180 min.), the green light input was perturbed by subtracting 50% of its intensity. The gains used for achieving the setpoint during the first phase were insufficient to bring the system back to the setpoint in a reasonable amount of time. The controller reacted too slowly to the error generated by the perturbation; the green light input applied between minutes 300 to 400 was almost constant. At the same time, the two-component system used responds very weakly and slowly to small changes in the green light intensity. To overcome this problem, the PI gains were switched at $t = 410$ min. (dashed vertical black line) to make the controller more "aggressive". Since the system was away from the setpoint, this resulted in oscillatory behavior. However, when the gain switch is done right after the setpoint has been achieved, disturbances are nicely rejected. This so-called gain scheduling approach [2] was used on all disturbance rejection experiments which involved PI control (Fig.3a,c). More specifically, K_P was doubled (from 80 to 160) and K_I was increased from 8 to 20 as soon as the setpoint had been achieved. The system was left unperturbed for an additional hour before applying any perturbation to demonstrate that the gain scheduling did not affect setpoint tracking.

Supplementary Note 11. Dose-response characterization

Each point on the figure below corresponds to the measurement of the fold-change with respect to the initial mean normalized GFP fluorescence of a cell culture brought to steady-state under constant light intensity. The green line corresponds to a sigmoidal curve of the form

$$f(x) = \frac{5.134}{1 + 5.411e^{-0.0698x}} + 0.1992$$

fitted to the experimental data. Day-to-day variability is manifested in the differences across steady-state levels for the same light intensity (experiments done on different days).

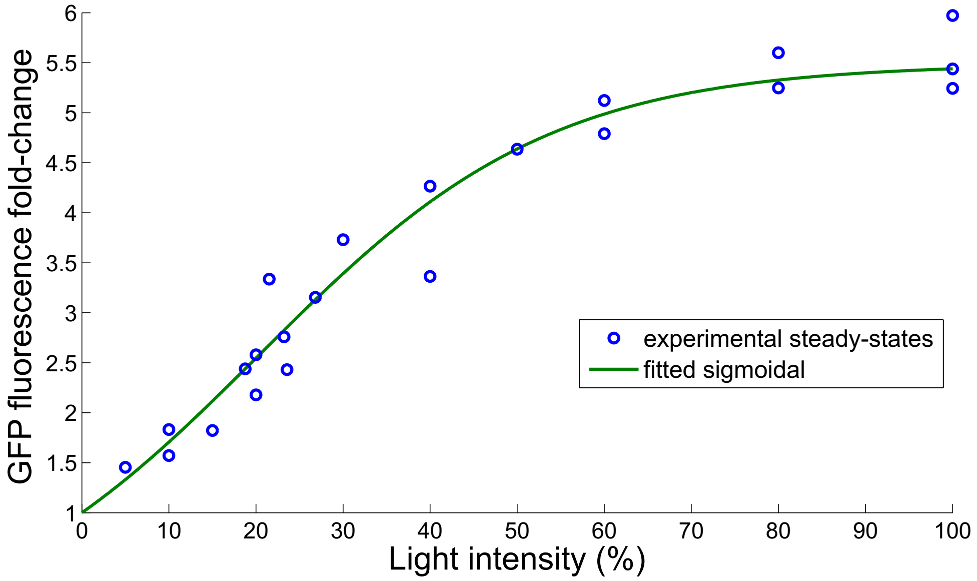

Figure S 11.1: Steady-state normalized GFP fold-change as a function of green LED intensity.

Supplementary Note 12. Derivation of discrete-time model equations

The differential equations for the fold-change system given in Online Methods have the following vector form:

$$\frac{dx}{dt} = A_c x + B_c \begin{bmatrix} 1 \\ u \end{bmatrix}$$
$$y = Cx$$

where $x = \begin{bmatrix} R & P & G \end{bmatrix}^T$ denotes the system state, $y = G$ the measured output and

$$A_c = \begin{bmatrix} -d_r & 0 & 0 \\ d_p + k_m & -d_p - k_m & 0 \\ 0 & d_p & -d_p \end{bmatrix}, \; B_c = \begin{bmatrix} d_r & b_r \\ 0 & 0 \\ 0 & 0 \end{bmatrix} \text{ and } C = \begin{bmatrix} 0 & 0 & 1 \end{bmatrix}.$$

This continuous-time model can be converted into discrete-time for a given sample time T_s to yield a set of difference equations of the form [3]

$$x_{n+1} = Ax_n + B \begin{bmatrix} 1 \\ u_n \end{bmatrix}$$
$$y_n = Cx_n,$$

where x_n, y_n and u_n is shorthand for $x(nT_s)$, $y(nT_s)$ and $u(nT_s)$, i.e. the state, output at n-th multiple of the sampling time and

$$A = e^{A_c T_s} \text{ and } B = A_c^{-1}(A - I)B_c.$$

The model form used in this work has two additional modifications: an input delay of one time step and an additional, uncontrolled input (d) that models the effect of additive disturbances. Thus, the final model equations are

$$x_{n+1} = Ax_n + B \begin{bmatrix} 1 \\ u_{n-1} + d_n \end{bmatrix}$$
$$y_n = Cx_n.$$

Supplementary Note 13. Particle filter for state and parameter estimation

General setup: As explained in Online Methods, the model described above is not sufficient to account for external perturbations that may alter the system dynamics, since matrices A and B are assumed fixed for all time steps. We therefore adopted a more flexible modeling approach, where system parameters are allowed to vary from one time step to the next. The increased flexibility, however, also creates the risk of over-fitting. This is counteracted by explicitly stating a set of assumptions regarding the allowed parameter changes from step to step (as well as the overall range of allowed parameter variations) and by properly balancing model fidelity with measurement accuracy during the estimation process.

All these features are naturally included in the recursive Bayesian state and parameter estimation framework [7], which we implemented in this work. Without getting into technical details, in this approach we assume that the system states and parameters are sequences of random variables generated by a Markov process. The goal is then to estimate the sequence of states and the system parameters based on a sequence of observed outputs and applied inputs. The so-called particle filters [4] are powerful Monte Carlo algorithms that implement this estimation procedure computationally.

More analytically, the evolution of states and parameters is assumed to be given by

$$\begin{aligned} x_{n+1} &= A(\theta_n)x_n + B(\theta_n)\begin{bmatrix} 1 \\ u_{n-1}+d_n \end{bmatrix} + w_n \\ \theta_{n+1} &= \theta_n + v_n, \end{aligned}$$

where $\theta = \begin{bmatrix} d_r & d_p & k_m & b_r & d \end{bmatrix}$ is the vector that contains all unknown system parameters, while $\{w_n\}$ and $\{v_n\}$ are Gaussian white noise processes, i.e. $w_n \sim \mathcal{N}(0, \Sigma_s)$ and $v_n \sim \mathcal{N}(0, \Sigma_p)$ for every n[1]. Both covariance matrices are diagonal (i.e. noise affects each component of the state and parameter vector independently). Note that the parameter vector contains the reaction rates of the original, continuous-time model, together with the unknown disturbance input. Once the continuous-time model parameters are assigned a numerical value, matrices A and B of the discrete-time model can be computed, as shown in Section 12

At each time step n, the measured output y_n^{meas} is assumed to be a noisy observation of $y_n = Cx_n$. That is,

$$y_n^{meas} = y_n + z_n,$$

where $z_n \sim \mathcal{N}(0, \sigma_{meas}^2)$.

Following the Bayesian approach, the parameter vector at $n = 0$ is assumed to be a 5-dimensional random variable with a given prior distribution $\mathcal{N}(\theta^{nom}, \Sigma_\theta)$, where θ^{nom} is the maximum-likelihood parameter set described in Online Methods and Σ_θ is a diagonal covariance matrix that reflects our *a priori* uncertainty about parameter values.

The system states are also assigned a prior distribution, but it is a degenerate one: all states are equal to 1 with certainty before any green light input is applied, since the cells have been growing in the dark.

The marginal particle filter: Particle filters operate with a set of samples (called particles) that are used to approximate the current state and parameter distributions given the sequence of past outputs and inputs to the system. More concretely, given the prior distributions of parameters and states, a sequence of output measurements from the beginning of the experiment

[1] $\mathcal{N}(\mu, \Sigma)$ denotes the Gaussian distribution with mean μ and covariance matrix Σ.

up to the current time step T, $\{y_n^{meas}\}_{n=1}^T$ and the sequence of the inputs applied so far, $\{u_n\}_{n=1}^T$, the filter generates sets of P particles $\{x_{p,n}\}_{p=1}^P$ and $\{\theta_{p,n}\}_{p=1}^P$ sequentially for all time steps $n = 1, \ldots, T$. The sets of particles $\{x_{p,T}\}_{p=1}^P$ and $\{\theta_{p,T-1}\}_{p=1}^P$ are used to represent the distribution of system states and parameters at the current time step T.

Below we provide a description of the specific particle filtering algorithm used here [5], that is adjusted to our modeling assumptions (Algorithm 1). The interested reader is directed to the literature for further information regarding particle filtering [4]. Note: $f(x|\mu, \sigma)$ denotes the Gaussian density with mean μ and variance σ^2, evaluated at point x.

Algorithm 1 Marginal particle filter.

```
{Setup particles}
Draw P parameter particles from the parameter prior: {θ_{p,0}}_{p=1}^P ~ N(θ^{nom}, Σ_θ); generate
    P state particles {x_{p,0}}_{p=1}^P, all equal to [1 1 1]^T.
for n from 1 to T do
    for p from 1 to P do
        {Propagate state particles}
        x_{p,n} = A(θ_{p,n-1}) x_{p,n-1} + B(θ_{p,n-1}) [1 ; u_{n-2} + d_{n-1}] + w_n,  w_n ~ N(0, Σ_s)
        y_{p,n}^{pred} = C x_{p,n}
    end for
    {Compute and normalize particle weights}
    w_p = f(Y_{p,n}^{pred} | y_n^{meas}, σ_{meas}^2)
    W_p = w_p / (Σ_{j=1}^{P} w_j)
    {Resample states and parameters}
    Draw P state particles from {x_{p,n}} and P parameter particles from {θ_{p,n-1}} according to
        weights {W_p}
    for p from 1 to P do
        {Perturb parameter particles}
        θ_{p,n} = θ_{p,n-1} + v_n,  v_n ~ N(0, Σ_p)
    end for
end for
return Final particle populations, {x_{T,p}, p = 1, ..., P} and {θ_{T-1,p}, p = 1, ..., P}.
```

The above particle filtering algorithm is run every time an output measurement becomes available. The specific algorithm parametrization used is given below:

$$P = 5000, \ \Sigma_s = diag(\begin{bmatrix}0.01 & 0.01 & 0.01\end{bmatrix}),$$

$$\Sigma_p = 10^{-3} \cdot diag(\begin{bmatrix}0.2539 & 0.0127 & 0.0037 & 0.2587 & 0.025\end{bmatrix}), \ \sigma_{meas}^2 = 0.0025,$$

$$\Sigma_\theta = 10^{-3} \cdot diag(\begin{bmatrix}1.0155 & 0.0509 & 0.0150 & 1.0347 & 0.1\end{bmatrix}).$$

Supplementary Note 14. Model Predictive Controller (MPC)

We first repeat the basic MPC loop, outlined in the main text:

Algorithm 2 A high-level description of MPC.

1: *Given*: a system model, the total number of time steps (N), a reference trajectory ($\{Y_n\}_{n=1}^N$), input constraints, a control horizon (H), cost function (F), initial state (x_0)
2: Set $n = 1$
3: **while** $n \leq N$ **do**
4: Determine input sequence that optimizes the cost function over the control horizon and respects the input constraints
5: Apply the first element of the input sequence
6: Wait for one sampling period. When new measurement becomes available, estimate the new system state
7: Shift the control horizon one step ahead, $n \leftarrow n + 1$
8: **end while**

The above procedure is summarized graphically on Figure S 14.1.

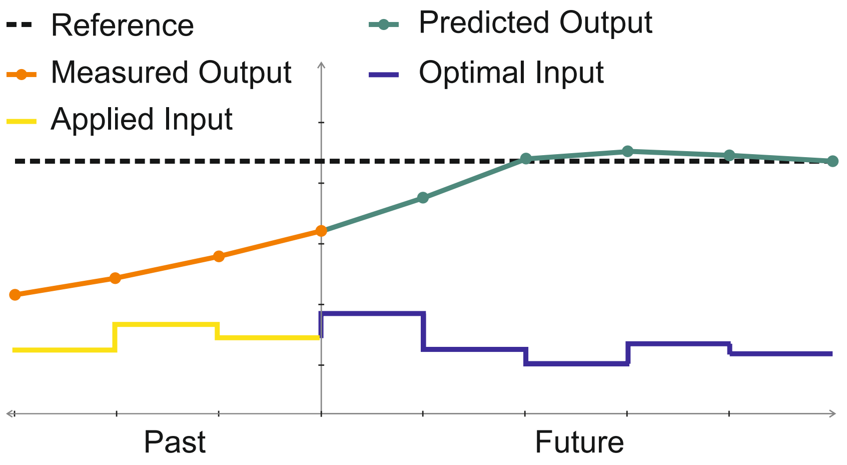

Figure S 14.1:

We now specify the details of the steps outlined above.

Controlled system: The system we control has the form

$$x_{n+1} = A(\theta)x_n + B(\theta)\begin{bmatrix}1\\u_n\end{bmatrix}$$
$$y_n = Cx_n,$$

Given the state x_n and parameter vector θ at the current time step n, together with an input sequence, $\{u_k\}_{k=n-1}^{n+H}$, the system can be simulated into the future to yield $\{x_k\}_{k=n}^{n+H}$ and $\{y_k\}_{k=n}^{n+H}$. Since knowledge of future parameter values is impossible, the parametrization of the model is assumed to be constant over time and fixed at the currently available value (θ).

Taking into account the fact that the particle filter operates with a set of joint state and parameter samples, there is not one but P alternative initial states and parameters at time n.

All these particles are thus simulated forward in time for a given input sequence. This result in the creation of a *distribution* of state and output trajectories, that depends on the current uncertainty about the system state and parametrization, as well as the way this is propagated through the system dynamics.

Reference trajectory: The reference trajectory is defined over the whole experiment timespan (i.e. step 0 to N). It is given by a set of values $\{Y_n\}_{n=0}^{N}$.

Input constraints: Since the system input is assumed to represent steady-state GFP fold changes, it can only take non-negative values. On the other hand, we know that the maximum GFP fold change achievable by this system is a little above 5. For this reason, the input u needs to be limited at 4 (recall that u denotes the *additional* GFP fold change above un-induced conditions). It therefore must hold $0 \leq u_n \leq 4,\ n = 0, \ldots, N$.

Cost function: The cost function (F) quantifies the aggregate deviation of the P output trajectories obtained under a given input sequence $\overline{u}_n = \{u_k\}_{k=n-1}^{n+H}$ (with u_{n-1} fixed from a previous optimization round) from the reference trajectory over the control horizon H. To define F we first compute for each particle $(x_{p,n}, \theta_{p,n})$ the deviation of the output trajectory from the reference, denoted by $F_p,\ p = 1, \ldots, P$:

$$F_p(x_{p,n}, \overline{u}_n) = \sum_{k=n}^{n+H} (Y_k - y_{p,k})^2.$$

We then reject all F_p values greater than the 90% quantile, as well as all values smaller than the 10% quantile and average the remaining $F_p(\overline{u}_n)$ values to obtain $F(\overline{u}_n)$. In this way, $F(\overline{u}_n)$ can be thought of as the "average" quadratic deviation of model predictions from the reference for a given input sequence.

$F(\overline{u}_n)$ is in turn optimized over all non-negative input sequences of length H with the help of the `fmincon` Matlab function, using an active-set method for constrained nonlinear optimization (Sequential Quadratic Programming).

Supplementary Methods

Construction of strains and plasmids for growth rate control experiments.

pZE3-*$P_{cpcG2\Delta59\_RBS3}$*-*gfpmut3*-*P_{ccaR_RBS}*-*ccaR::FLAG-ccaS::FLAG* (pSKA413): The pZE3 backbone [8] was amplified from pJT119b [6] using primers 240 and 392. *gfpmut3* [9] was amplified from DL4259 genomic DNA [10] using primers 598 and 393. *ccaR* was amplified from pJT119b and tagged with a C-terminal FLAG tag (DYKDDDDK) using primers 451 and 304. The *ccaR::FLAG* product was then reamplified with primers 451 and 394. *ccaS* was amplified from pJT119b and tagged with a C-terminal FLAG tag using primers 235 and 236. The *ccaS::FLAG* product was then reamplified with primers 235 and 395. A variant of the *cpcG2* promoter with 59 bp of a putative constitutive promoter deleted ($P_{cpcG2\Delta59}$, Supplementary Figure 1, and ref. [11]), a synthetic *gfpmut3* RBS3 (AAGGGACAGGCACGTAAGGCACCATATA, 467.61 Translation Initiation Rate, RBS Library Calculator [12]) and an AGGA *ccaR* RBS was amplified from pJT119b using primers 459 and 597. The resulting PCR products were Gibson assembled [13] and cloned into NEB Turbo (New England Biolabs) to construct pSKA413 (Supplementary Figure 1 and 2, Addgene plasmid ID #80381).

JT2 *ΔmetE::FRT Tn7::FRT*-*$P_{cpcG2\Delta59}$*-*metE* (SKA974):
<u>Deletion of *metE* from *E. coli* JT2</u>
The kanamycin-resistance cassette in pKD13 [14] was converted to chloramphenicol-resistance by amplifying the pKD13 backbone with primers 514 and 565 and amplifying chloramphenicol acetyltransferase (*cat*) from pKD3 [14] with primers 566 and 338. The PCR products were Gibson assembled and cloned into CC118(λpir) [15] to construct pSKA373.

To delete *metE* from strain JT2 [16], pSKA373 was amplified with primers 558 and 559 [17]. The PCR product was recombineered into JT2 using pKOBEGA as previously described [18] to construct JT2 Δ*metE::cat* (SKA932). The chloramphenicol-resistance cassette was then excised from strain SKA932 using FLP-recombinase plasmid pCP20 as previously described [19] to construct JT2 Δ*metE::FRT* (SKA936).

<u>Tn7 integration of $P_{cpcG2\Delta59}$-*metE* into JT2 Δ*metE::FRT*</u>
The kanamycin-resistance cassette in pUC18R6KT-mini-Tn7T [20] was converted to chloramphenicol-resistance by amplifying the FRT-flanked chloramphenicol-resistance cassette from pSKA373 using primers 84 and 85. The PCR product was ligated into pUC18R6KT-mini-Tn7T between SalI and NheI and cloned into CC118(λpir) [15] to construct pSKA385.

metE from MG1655 (ATCC 47076) and an *rrnBT1T2* transcriptional terminator from pBAD33 [21] was amplified from pSKA374 (pZE1-P_{tac}-*metE-rrnBT1T2*, unpublished, sequence available upon request) using primers 551 and 333. The *cpcG2* promoter was amplified from pJT119b using primers 536 and 344. The PCR products were Gibson assembled [13] into pSKA385 between SpeI and XhoI and cloned into CC118(λpir) [15] to construct pSKA396.

A variant of the *cpcG2* promoter with 59 bp of a putative constitutive promoter deleted ($P_{cpcG2\Delta59}$, Supplementary Figure 1 and ref. [11]) was amplified from pJT119b [6] using primers 536 and 449. The 5' end of *metE* was amplified from pSKA396 using primers 461 and 587. The PCR products were Gibson assembled [13] into

pSKA396 between SpeI and DraIII and cloned into CC118(λpir) [15] to construct pSKA397 (Supplementary Figure 1 and 2, Addgene plasmid ID #80380).

Integration plasmid pSKA397 and Tn7 transposase helper plasmid pTNS3 [22] were transformed into MFDpir [23] and cells were maintained by adding diaminopimelic acid (100 μg/ml) to the media. MFDpir pSKA397 and MFDpir pTNS3 were used to integrate *cat-*$P_{cpcG2\Delta 59}$*-metE* into the chromosome of SKA936 using tri-parental mating [22], [23]. Exconjugants JT2 Δ*metE::FRT Tn7::cat-*$P_{cpcG2\Delta 59}$*-metE* (SKA973) were selected for on LB-kan-cam agar and Tn7 integration was verified by sequencing. The chloramphenicol-resistance cassette was excised from strain SKA936 *Tn7::cat-*$P_{cpcG2\Delta 59}$*-metE* using FLP-recombinase plasmid pCP20 as previously described [19] to construct JT2 Δ*metE::FRT Tn7::FRT-*$P_{cpcG2\Delta 59}$*-metE* (SKA974, Addgene bacterial strain ID #80403).

Plasmids pPLPCB(S) [16] and pSKA413 were transformed into SKA974. The resulting strain was used for all growth rate control experiments.

Bibliography

[1] K. J. Astrom, “PID controllers: theory, design and tuning,” *Instrum. Soc. Am.*, 1995.

[2] K. J. Åström and T. Hägglund, *Advanced PID control*. ISA-The Instrumentation, Systems, and Automation Society; Research Triangle Park, NC 27709, 2006.

[3] C.-T. Chen, *Linear system theory and design*. Oxford University Press, Inc., 1995.

[4] A. Doucet, N. De Freitas, and N. J. Gordon, “Sequential Monte Carlo Methods in Practice. Series Statistics For Engineering and Information Science.” Springer New York, 2001.

[5] M. Klaas, N. De Freitas, and A. Doucet, “Toward practical N2 Monte Carlo: The marginal particle filter,” *arXiv Prepr. arXiv1207.1396*, 2012.

[6] E. J. Olson, L. a Hartsough, B. P. Landry, R. Shroff, and J. J. Tabor, “Characterizing bacterial gene circuit dynamics with optically programmed gene expression signals.,” *Nat. Methods*, vol. 11, no. August 2013, pp. 449–55, 2014.

[7] S. Särkkä, *Bayesian filtering and smoothing*, vol. 3. Cambridge University Press, 2013.

[8] R. Lutz and H. Bujard, “Independent and Tight Regulation of Transcriptional Units in Escherichia Coli Via the LacR/O, the TetR/O and AraC/I1-I2 Regulatory Elements,” *Nucleic Acids Res.* , vol. 25 , no. 6 , pp. 1203–1210, Mar. 1997.

[9] B. P. Cormack, R. H. Valdivia, and S. Falkow, “FACS-optimized mutants of the green fluorescent protein (GFP),” *Gene*, vol. 173, no. 1, pp. 33–38, 1996.

[10] R. P. Morse, K. C. Nikolakakis, J. L. E. Willett, E. Gerrick, D. A. Low, C. S. Hayes, and C. W. Goulding, “Structural basis of toxicity and immunity in contact-dependent growth inhibition (CDI) systems,” *Proc. Natl. Acad. Sci.* , vol. 109 , no. 52 , pp. 21480–21485, Dec. 2012.

[11] S. R. Schmidl, R. U. Sheth, A. Wu, and J. Tabor, “Refactoring and Optimization of Light-Switchable Escherichia coli Two-Component Systems,” 2014.

[12] I. Farasat, M. Kushwaha, J. Collens, M. Easterbrook, M. Guido, and H. M. Salis, “Efficient search, mapping, and optimization of multi- protein genetic systems in diverse bacteria,” *Mol. Syst. Biol.*, vol. 10, no. 6, Jun. 2014.

[13] D. G. Gibson, L. Young, R.-Y. Chuang, J. C. Venter, C. A. Hutchison, and H. O. Smith, “Enzymatic assembly of DNA molecules up to several hundred kilobases,” *Nat. Methods*, vol. 6, no. 5, pp. 343–345, 2009.

[14] K. A. Datsenko and B. L. Wanner, “One-step inactivation of chromosomal genes in Escherichia coli K-12 using PCR products,” *Proc. Natl. Acad. Sci.* , vol. 97 , no. 12 , pp. 6640–6645, Jun. 2000.

[15] M. Herrero, V. de Lorenzo, and K. N. Timmis, “Transposon vectors containing non-antibiotic resistance selection markers for cloning and stable chromosomal insertion of foreign genes in gram-negative bacteria.,” *J. Bacteriol.* , vol. 172 , no. 11 , pp. 6557–6567, Nov. 1990.

[16] J. J. Tabor, A. Levskaya, and C. a Voigt, “Multichromatic control of gene expression in Escherichia coli. Supplemental,” *J. Mol. Biol.*, vol. 405, pp. 315–24, 2011.

[17] T. Baba, T. Ara, M. Hasegawa, Y. Takai, Y. Okumura, M. Baba, K. A. Datsenko, M. Tomita, B. L. Wanner, and H. Mori, “Construction of Escherichia coli K- 12 in- frame, single- gene knockout mutants: the Keio collection,” *Mol. Syst. Biol.*, vol. 2, no. 1, Feb. 2006.

[18] A. Roux, C. Beloin, and J.-M. Ghigo, “Combined Inactivation and Expression Strategy To Study Gene Function under Physiological Conditions: Application

to Identification of New Escherichia coli Adhesins," *J. Bacteriol.* , vol. 187 , no. 3 , pp. 1001–1013, Feb. 2005.
[19] P. P. Cherepanov and W. Wackernagel, "Gene disruption in Escherichia coli: TcR and KmR cassettes with the option of Flp-catalyzed excision of the antibiotic-resistance determinant," *Gene*, vol. 158, no. 1, pp. 9–14, 1995.
[20] K.-H. Choi, J. B. Gaynor, K. G. White, C. Lopez, C. M. Bosio, R. R. Karkhoff-Schweizer, and H. P. Schweizer, "A Tn7-based broad-range bacterial cloning and expression system," *Nat Meth*, vol. 2, no. 6, pp. 443–448, Jun. 2005.
[21] L. M. Guzman, D. Belin, M. J. Carson, and J. Beckwith, "Tight regulation, modulation, and high-level expression by vectors containing the arabinose PBAD promoter.," *J. Bacteriol.* , vol. 177 , no. 14 , pp. 4121–4130, Jul. 1995.
[22] K.-H. Choi, T. Mima, Y. Casart, D. Rholl, A. Kumar, I. R. Beacham, and H. P. Schweizer, "Genetic Tools for Select-Agent-Compliant Manipulation of Burkholderia pseudomallei ," *Appl. Environ. Microbiol.* , vol. 74 , no. 4 , pp. 1064–1075, Feb. 2008.
[23] L. Ferrières, G. Hémery, T. Nham, A.-M. Guérout, D. Mazel, C. Beloin, and J.-M. Ghigo, "Silent Mischief: Bacteriophage Mu Insertions Contaminate Products of Escherichia coli Random Mutagenesis Performed Using Suicidal Transposon Delivery Plasmids Mobilized by Broad-Host-Range RP4 Conjugative Machinery ," *J. Bacteriol.* , vol. 192 , no. 24 , pp. 6418–6427, Dec. 2010.
